# *Candida albicans’* inorganic phosphate transport and evolutionary adaptation to phosphate scarcity

**DOI:** 10.1371/journal.pgen.1011156

**Published:** 2024-08-13

**Authors:** Maikel Acosta-Zaldívar, Wanjun Qi, Abhishek Mishra, Udita Roy, William R. King, Yuping Li, Jana Patton-Vogt, Matthew Z. Anderson, Julia R. Köhler

**Affiliations:** 1 Division of Infectious Diseases, Boston Children’s Hospital/Harvard Medical School, Boston, Massachusetts, United States of America; 2 Center for Genomic Science Innovation, University of Wisconsin-Madison, Madison, Wisconsin, United States of America; 3 Department of Biological Sciences, Duquesne University, Pittsburgh, Pennsylvania, United States of America; 4 Department of Microbiology and Immunology, University of California, San Francisco, California, United States of America; 5 Department of Medical Genetics, Laboratory of Genetics, University of Wisconsin-Madison, Madison, Wisconsin, United States of America; CAU: Christian-Albrechts-Universitat zu Kiel, GERMANY

## Abstract

Phosphorus is essential in all cells’ structural, metabolic and regulatory functions. For fungal cells that import inorganic phosphate (Pi) up a steep concentration gradient, surface Pi transporters are critical capacitators of growth. Fungi must deploy Pi transporters that enable optimal Pi uptake in pH and Pi concentration ranges prevalent in their environments. Single, triple and quadruple mutants were used to characterize the four Pi transporters we identified for the human fungal pathogen *Candida albicans*, which must adapt to alkaline conditions during invasion of the host bloodstream and deep organs. A high-affinity Pi transporter, Pho84, was most efficient across the widest pH range while another, Pho89, showed high-affinity characteristics only within one pH unit of neutral. Two low-affinity Pi transporters, Pho87 and Fgr2, were active only in acidic conditions. Only Pho84 among the Pi transporters was clearly required in previously identified Pi-related functions including Target of Rapamycin Complex 1 signaling, oxidative stress resistance and hyphal growth. We used in vitro evolution and whole genome sequencing as an unbiased forward genetic approach to probe adaptation to prolonged Pi scarcity of two quadruple mutant lineages lacking all 4 Pi transporters. Lineage-specific genomic changes corresponded to divergent success of the two lineages in fitness recovery during Pi limitation. Initial, large-scale genomic alterations like aneuploidies and loss of heterozygosity eventually resolved, as populations gained small-scale mutations. Severity of some phenotypes linked to Pi starvation, like cell wall stress hypersensitivity, decreased in parallel to evolving populations’ fitness recovery in Pi scarcity, while severity of others like membrane stress responses diverged from Pi scarcity fitness. Among preliminary candidate genes for contributors to fitness recovery, those with links to TORC1 were overrepresented. Since Pi homeostasis differs substantially between fungi and humans, adaptive processes to Pi deprivation may harbor small-molecule targets that impact fungal growth, stress resistance and virulence.

## Author summary

Fungi must be able to access enough phosphate in order to invade the human body. Virulence of *Candida albicans*, the most common invasive human fungal pathogen, is known to decrease when one of the proteins that brings phosphate into the fungal cell, called Pho84, is disabled. We identified three more proteins in *C*. *albicans* that transport phosphate into the cell. We found that Pho84 plays the largest role among them across the broadest range of environmental conditions. After eliminating all 4 of these transporters, we let two resulting mutants evolve for two months in limited phosphate and analyzed the growth and stress resistance of the resulting populations. We analyzed genomes of representative populations and found that early adaptations to phosphate scarcity occurred together with major changes to chromosome configurations. In later stages of the adaptation process, these large-scale changes disappeared as populations gained small-scale mutations that increased cells’ ability to grow in limited phosphate. Some but not all of these favorable mutations improved resistance of evolving populations to stressors like membrane- and cell wall stress. Pinpointing distinct mutation combinations that affect stress resistance differently in populations adapting to scarce phosphate, may identify useful antifungal drug targets.

## Introduction

Phosphorus is an essential macronutrient for living cells and a major component of chromosomes, membranes and the transcription and translation machineries [1]. Inorganic phosphate (Pi) is required in the production of ATP, the energy currency of the cell, that governs central metabolic processes and intracellular signaling. Consequently, Pi is not only required for growth and proliferation but also for survival: e.g., fission yeast cells starved for Pi initially become quiescent and then lose viability [2].

Osmotrophic organisms that take up soluble small-molecule nutrients from their immediate environment must import Pi separately from molecular sources of nitrogen and carbon to acquire sufficient phosphorus. Most soils and aquatic environments contain <1%, or ≤10 mM soluble Pi, so that Pi is a scarce resource for plants and free-living microorganisms [3–5]; human serum Pi ranges from 0.8–1.3 mM [6], suggesting that microbial invasive pathogens of humans can also experience Pi deprivation. Ausukaree et al. used ^31^P NMR spectroscopy to determine that the *S*. *cerevisiae* PHO regulon is tuned to maintain intracellular free- and polyphosphate Pi at ∼25 and 210 mM, respectively [7]; we found that *C*. *albicans’* intracellular Pi concentrations are in similar ranges as *S*. *cerevisiae’s* but vary more widely in conditions of extracellular low or high Pi [8]. The fundamental challenge for these organisms therefore is the steep concentration gradient between intracellular and extracellular Pi concentrations. For this reason, the Pi-homeostatic systems of small-molecule importing organisms like bacteria, plants and fungi have much in common. Orthology of phosphate-proton symporters among plants and fungi was first determined by complementation of a *Saccharomyces cerevisiae* null mutant in *PHO84* with two *Arabidopsis thaliana* Pi transporters [9,10]. In contrast, human phosphate homeostasis regulation differs fundamentally from that of osmotrophs [11,12], and since abundant phosphorus-containing molecules are present in all human food sources of protein, the major high-affinity Pi transporter of fungi has no human homolog.

*Saccharomyces cerevisiae* Pi transporters were characterized over decades according to their Pi affinity and their pH optima [13]. Kinetic studies in the 1980s identified two Pi transport systems, one with a low K_m_ value of 8.4–21.4 μM, defined by the early investigators of these systems as high-affinity, and another with a high K_m_ value of 0.77–1.7 mM, defined as low-affinity [14,15]. Further analysis suggested two separate transporters with distinct pH optima within the high-affinity uptake system [16]. Cloning and functional characterization of the *PHO84* gene showed that its product is a component of the high-affinity Pi transport system [17]. Heterologous expression of Pho84 and its incorporation into liposomes then permitted kinetic studies that demonstrated a K_m_ for Pi of 24 μM [18]. *S*. *cerevisiae* Pho84 is a member of the Phosphate: H^+^ Symporter Family within the Major Facilitator Superfamily [19]; it uses the chemiosmotic energy of proton symport to transport the Pi anion up a concentration gradient across the plasma membrane.

Additional Pi import systems were subsequently identified and characterized. A separate high-affinity Pi uptake system that was enhanced in the presence of sodium at pH 7.2 was described [20]; the gene encoding this activity was later identified and its product, named Pho89, confirmed to have a Pi K_m_ of 0.5 μM [16]. Pho89 belongs to solute carrier family 20 as a sodium-dependent phosphate transporter [21,22]. Low-affinity *S*. *cerevisiae* Pi transporters Pho87, Pho90 and Pho91 were subsequently characterized genetically and functionally [23]. Pho91 was later shown to reside on the vacuolar membrane and facilitate Pi export from the vacuole to the cytosol [24]. Further work showed distinct activities of the 2 low-affinity transporters: Pho87 versus Pho90 can sustain growth of *S*. *cerevisiae* down to Pi concentrations of 5 mM versus 0.5 mM, respectively [25]. *S*. *cerevisiae* therefore has 2 high-affinity Pi transporters, Pho84 and Pho89, whose energetic drivers are proton- and sodium symport, respectively, and 2 paralogous low-affinity Pi transporters, Pho87 and Pho90 [23,25].

The genome of the opportunistic fungal pathogen *Candida albicans* encodes 4 homologs of *S*. *cerevisiae* Pi transporters. In a *mariner* transposon mutant screen we previously identified a mutant in the *C*. *albicans* homolog of *S*. *cerevisiae PHO84* as hypersensitive to rapamycin [8]. We showed that *C*. *albicans PHO84* is required in normal Target of Rapamycin Complex 1 (TORC1) signaling, oxidative- and cell wall stress resistance, survival during exposure to amphotericin B and the echinocandin micafungin, and normal virulence [8,26,27]. Given the presence of other Pi transporter homologs in the *C*. *albicans* genome, we sought to understand how loss of just one, Pho84, could significantly impact important physiological functions and even virulence in *C*. *albicans*.

Pi-acquisition and -homeostatic systems (PHO regulons) of bacterial and other fungal human pathogens are required for virulence, implicating Pi scarcity as a prevalent condition in the host [28–31]: e.g., in a pathogen that is completely adapted to its human host, *Mycobacterium tuberculosis*, transcription of a secretion system for virulence factors is activated by Pi starvation [32,33]. Expression of high-affinity Pi transporters is typically regulated according to ambient Pi concentrations [34]. In *S*. *cerevisiae*, high-affinity Pi transporter-encoding genes *PHO84* and *PHO89* are upregulated during Pi starvation [23,35,36]. In *C*. *albicans* ex vivo and in vivo infection models [37–41], the *PHO84* and *PHO89* homologs are similarly upregulated [16]. These findings suggest that in the host, *C*. *albicans* like *M*. *tuberculosis* experiences Pi starvation. In another important fungal pathogen, *Cryptococcus neoformans*, the transcriptional regulator Pho4 that upregulates Pi acquisition systems during Pi starvation plays a critical role in virulence: *C*. *neoformans* strains that lack Pho4 are profoundly attenuated in virulence in two distinct mouse models of cryptococcal infection [42]. Given the importance of Pi in *C*. *albicans’* pathogenetic process, we set out to identify and characterize the putative *C*. *albicans* Pi importers other than Pho84 that can contribute to cytosolic Pi availability for the fungus’ growth and interaction with the host [8,26,27,43].

We then asked whether *C*. *albicans* can adapt to persistent Pi scarcity. Forward genetic screens of chemically or transposon-mutagenized cells subjected to specific selective conditions are powerful discovery tools because they provide unbiased and often unexpected information [44]. In vitro evolution and whole genome analysis has been used in other pathogens for the characterization of drug responses [45–49] and in *Candida* species for analysis of drug resistance development [50–54]. We questioned whether this approach might have benefits for analysis of adaptation to nutrient scarcity compared with mutant screens. Its possible advantages might be a higher likelihood of revealing illuminating gain-of-function mutations and the ability to uncover mutations involved in polygenic traits. We used in vitro evolution and genome analysis to begin uncovering the cellular processes linked to *C*. *albicans’* management of Pi scarcity. Preliminary analysis of candidate genes contributing to fitness recovery during evolution in Pi scarcity revealed several genes linked to TORC1 signaling.

## Results

### *PHO84* plays a central role in growth and filamentation

Homology searches identified four Pi transporters in the *C*. *albicans* genome including *PHO84*. A *C*. *albicans* ortholog of *S*. *cerevisiae PHO89*, which encodes a high-affinity Pi transporter with an alkaline optimum [55], resides on chromosome 4. Homology searches using the low affinity *S*. *cerevisiae PHO87* and *PHO90* paralogs found a single homolog named *PHO87* in the Candida Genome Database (CGD) [55]. A *PHO84* homolog named *FGR2* was also identified that shares 23% identity and 42% similarity [56] of the amino acid sequence with Pho84 (Fig A in S1 Text). A screen of heterozygous *C*. *albicans* mutants first found *FGR2* to be involved in filamentous growth [57] and more recently it was noted to contribute to filamentation differences between *C*. *albicans* strains [58]. To delineate the contribution of these 4 predicted transporters to Pi acquisition, we constructed single gene deletion mutants for *PHO89*, *PHO87* and *FGR2* using our FLP-*NAT1* system [59] to compare with our previously constructed *pho84*-/- mutants [8].

**Fig 1:**
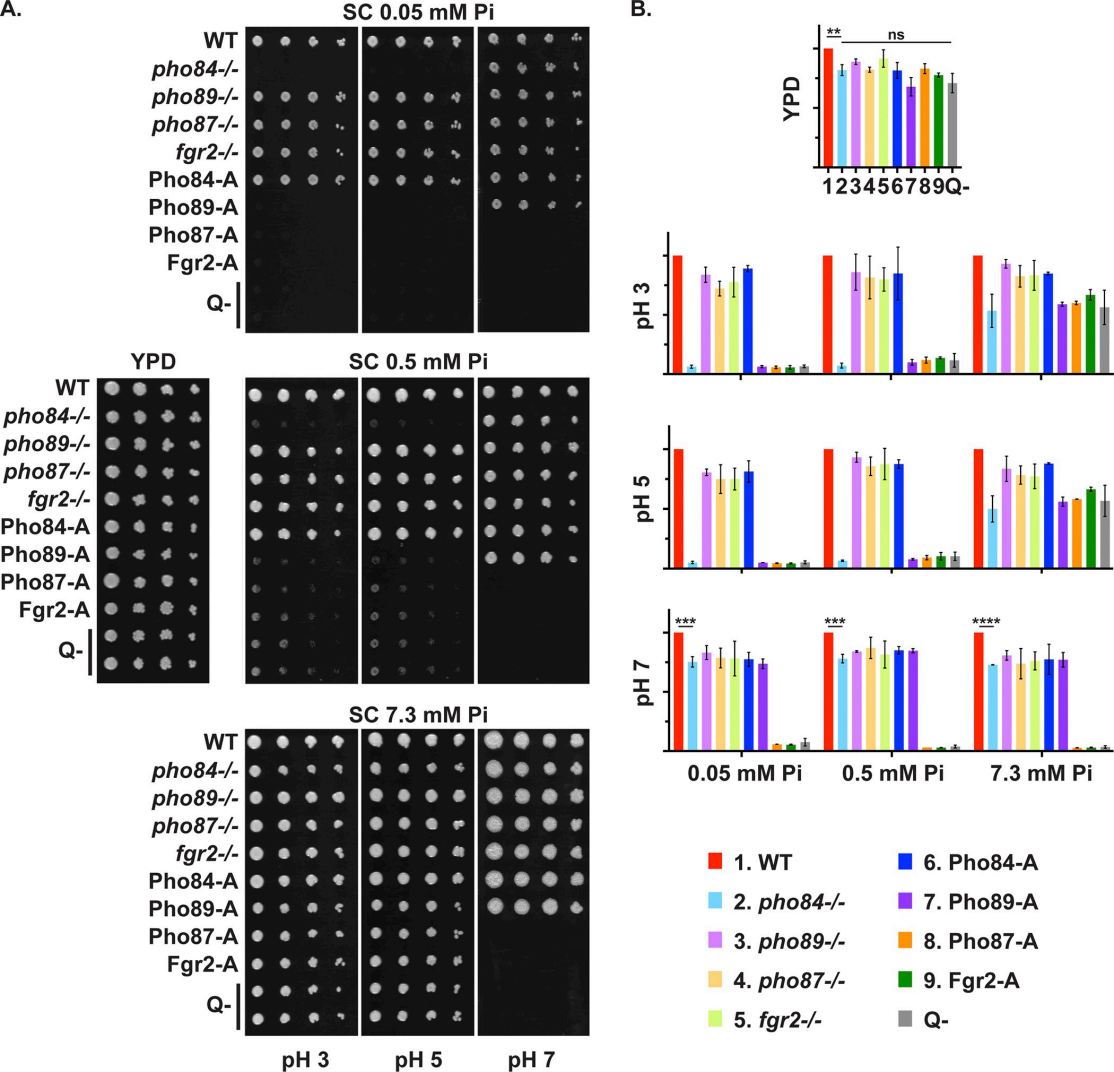
Among Pi transporters, Pho84 contributed to growth over the broadest range of tested conditions. **A.** Fivefold dilutions of cells of indicated genotypes were spotted (left to right) onto YPD (left, center) or SC (all others) agar media buffered to indicated pH (3, 5 or 7) and containing indicated Pi concentrations (0.05, 0.5 or 7.3 mM) and grown at 30°C for 2 d. Strains are WT (JKC915); *pho84*-/- (JKC1450); *pho89*-/- (JKC2585); *pho87*-/- (JKC2581); *fgr2*-/- (JKC2667); Pho84-A: *pho87*-/- *pho89*-/- *fgr2*-/- *PHO84*+/+ (JKC2788); Pho89-A: *pho84*-/- pho87-/- *fgr2*-/- *PHO89*+/+ (JKC2783); Pho87-A: *pho84*-/- *pho89*-/- *fgr2*-/- *PHO87*+/+ (JKC2777); Fgr2-A: *pho84*-/- *pho87*-/- *pho89*-/- *FGR2*+/+ (JKC2758); Q-: *pho84*-/- *pho87*-/- *pho89*-/- *fgr2*-/- (JKC2830 and JKC2860). Representative of 3 biological replicates. **B.** Relative growth (normalized to WT) of strains with indicated genotypes under varying Pi availability and pH ranges. Spots’ integrated signal intensity ratio of each strain vs. WT control on the same plate was calculated and plotted in Graphpad Prism. WT, *pho84*-/-, *pho89*-/-, *pho87*-/-, Q- strains error bars represent SD from 3 biological replicates and Pho84-A, Pho89-A, Pho87-A strains error bars represent SD from 2 biological replicates. For statistical significance: ns, *p* > 0.05; **, 0.001 < *p* ≤ 0.01; ***, 0.0001 < *p* ≤ 0.001. At pH 3 and pH 5, no statistically significant difference was noted between growth of *pho84*-/-, and that of Pho89-A, Pho87-A, Fgr2-A and Q- cells.

Pi restriction reduced growth only of *pho84*-/- mutants at acidic pH (Fig 1). Null mutants in *PHO84* were unable to grow on synthetic complete (SC) agar medium containing low (0.05 mM) or moderate (0.5 mM) Pi at pH 3 or 5. In contrast, null mutants in the 3 other predicted Pi transporters grew similarly to the wildtype control strain (WT) under these conditions, indicating that only *PHO84* is required in moderate to low Pi at lower than neutral pH (Fig 1; shown in Fig 1 are also triple and quadruple mutants, which retain only one or none of the 4 Pi transporters, respectively; these are described below in detail). At pH 7, *pho84*-/- mutants grew at all tested Pi concentrations, indicating that one or more other Pi transporters were able to uptake sufficient Pi to sustain growth at neutral pH. At 7.3 mM Pi, all single mutants in the 4 predicted Pi transporters grew robustly, indicating that no single transporter is indispensable at high Pi concentrations (Fig 1).

**Fig 2:**
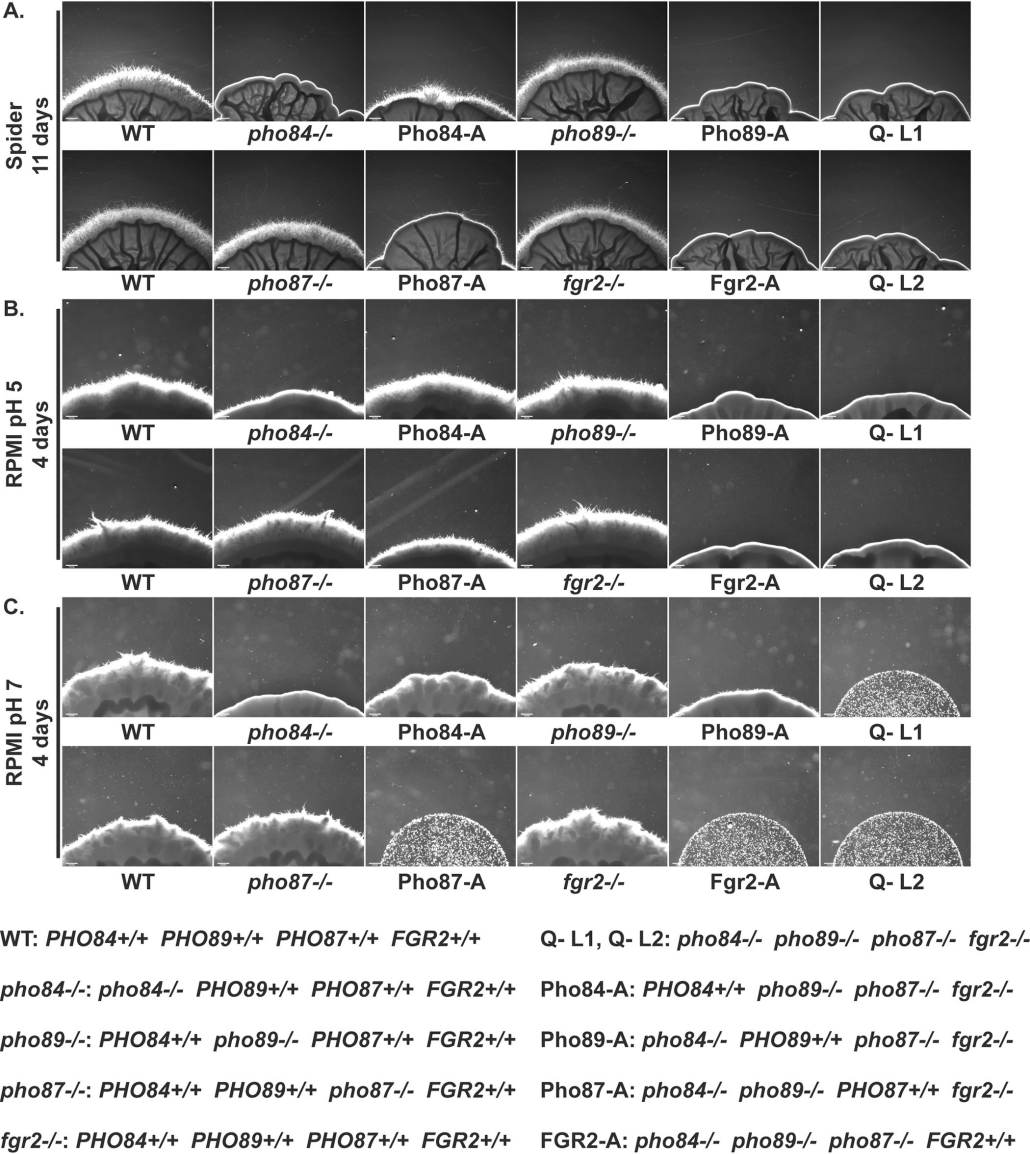
Pho84 was the major contributor to hyphal growth among 4 Pi transporters. Cell suspensions of indicated genotypes were spotted at equidistant points around the perimeter of Spider (**A**) and RPMI (**B, C**) agar plates. Photomicrographs of the edge of spots were obtained at 4 days for RPMI and 11 days for Spider plates. Spot edges were aligned with image frame corners to allow comparisons of hyphal fringes’ length. Spots without filaments have smooth rounded edges while hyphal growth appears like dense fuzz with spiky tips. Strains are WT (JKC915); *pho84-/-* (JKC1450); Pho84-A: *pho87-/- pho89-/- fgr2-/- PHO84+/+* (JKC2788); *pho89-/-* (JKC2585); Pho89-A: *pho84-/- pho87-/- fgr2-/- PHO89+/+* (JKC2783)*; pho87-/-* (JKC2581); Pho87-A: *pho84-/- pho89-/- fgr2-/- PHO87+/+* (JKC2777); *fgr2-/-* (JKC2667); Fgr2-A: *pho84-/- pho87-/- pho89-/- FGR2+/+* (JKC2758); Q- L1: *pho84-/- pho87-/- pho89-/- fgr2-/-* (JKC2830); Q- L2: *pho84-/- pho87-/- pho89-/- fgr2-/-* (JKC2860). Size bar 200 μm. Representative of 3 biological replicates.

*C*. *albicans’* ability to readily switch between growth as single budding yeast versus as multicellular filamentous hyphae contributes to its virulence [60,61]. Cells lacking *PHO84* were previously shown to be defective in hyphal formation [26,62]. To examine the role of the different Pi transporters in morphogenesis, cell suspensions from each of the 4 Pi transporter mutants were spotted on filamentation-inducing agar media. Most clearly on Spider medium, cells lacking *PHO84* had minimal or absent hyphal growth (Fig 2A). Single mutants of the other 3 Pi transporters showed more subtle filamentation defects than *pho84*-/- cells. Similar filamentation phenotypes were observed on RPMI agar at pH 5 and pH 7: *pho84-/-* mutants produced occasional thin wisps of peripheral hyphae only on RPMI at pH 5 but not pH 7, while *pho89-/-*, *pho87-/-* and *fgr2-/-* mutants showed robust hyphal growth on these media (Fig 2B and 2C). Together, these findings show that Pho84 is the most important predicted Pi transporter for filamentation under these conditions.

### Only *PHO84* impacted TORC1 signaling and oxidative stress endurance

**Fig 3:**
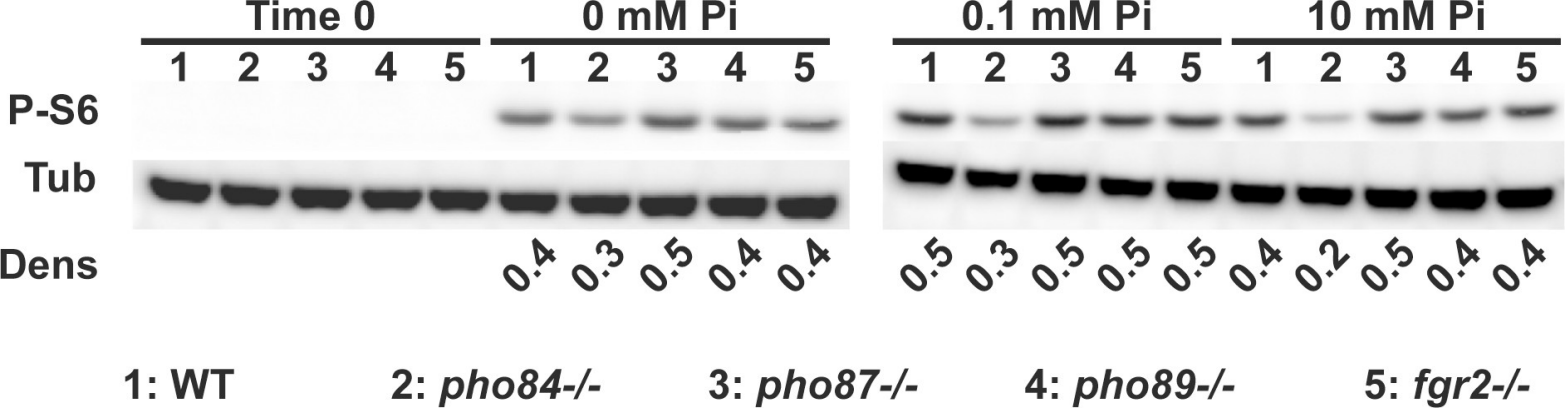
Pho84 was required for TORC1 activation. Cells were grown in YNB with indicated Pi concentrations for 90 min. Western blots were probed against phosphorylated Rps6 (P-S6) for monitoring TORC1 activity, and tubulin (Tub) as loading control. Dens: ratio between P-S6 and tubulin signals by densitometry. Representative of 3 biological replicates. Strains are 1: WT (JKC915); 2: *pho84-/-* (JKC1450); 3: *pho87-/-* (JKC2581); 4: *pho89-/-* (JKC2585) and 5: *fgr2-/-* (JKC2667).

We previously found that mutants in *PHO84* are hypersensitive to rapamycin and show decreased TORC1 signaling when growing in limited Pi [8], and that TORC1 co-regulates *PHO84* expression in addition to its known regulation by Pho4 [63,64]. TORC1 activation was reduced in *pho84-/-* cells as determined by the phosphorylation state of ribosomal protein S6 (P-S6), an established TORC1 activity readout [65] (Fig 3) while null mutants of the other Pi transporters did not show reduced P-S6. We concluded that Pho84 specifically contributes to TORC1 activation among the Pi transporters. TORC1 contributes to managing oxidative stress responses in *C*. *albicans*, and mutants in *PHO84* are known to be hypersensitive to oxidative stress [26,27,62]. Among single null mutants in each of the Pi transporters, only the *pho84*-/- mutant showed hypersensitivity to the superoxide inducer plumbagin (Fig B in S1 Text).

### Triple and quadruple mutants in predicted Pi transporters support a major role for Pho84

To examine the Pi uptake characteristics of each transporter, we constructed triple mutants that retained one predicted Pi transporter, Pho84, Pho89, Pho87 or Fgr2. Triple mutants retaining a single Pi transporter are abbreviated as the name of the sole remaining transporter followed by “Alone” or by a capital A for “alone among predicted Pi transporters” (e.g., Pho84-A is *pho89-/- pho87-/- fgr2-/-*). Among these triple mutants, only Pho84-Alone grew under all tested conditions (Fig 1). Under Pi limiting conditions, Pho89-Alone cells showed significant growth only at neutral pH (Fig 1). These results support a role for Pho89 as a high-affinity transporter with a more alkaline optimum as in *S*. *cerevisiae*. Pho87-Alone and Fgr2-Alone cells grew only in high Pi (7.3 mM) at acidic pH (pH 5 and pH 3) (Fig 1), suggesting that Pho87 and Fgr2 are low-affinity transporters with an acidic pH optimum.

We engineered two quadruple Pi transporter mutants (Q-, *pho84-/- pho89-/- pho87-/- fgr2-/-*) (please see “Strain construction” and “Construction of strains with multiple mutations” in Methods as well as S2–S4 Tables for details). These strains grew on rich complex medium, YPD, that contains organic phosphate compounds. They were then tested for growth on Pi as the sole phosphorus source at a range of concentrations. Q- cells were able to grow on high (7.3 mM) Pi at acidic pH, while on moderate (0.5 mM) Pi their growth was barely detectable (Fig 1). They did not grow at pH 7 or at a low Pi concentration (0.05 mM, Fig 1). These findings show that in cells lacking the 4 identified Pi transporters, a residual Pi transport capacity exists that is active at high Pi concentrations and at an acidic pH.

### *PHO84* supported growth under the broadest range of conditions

**Fig 4:**
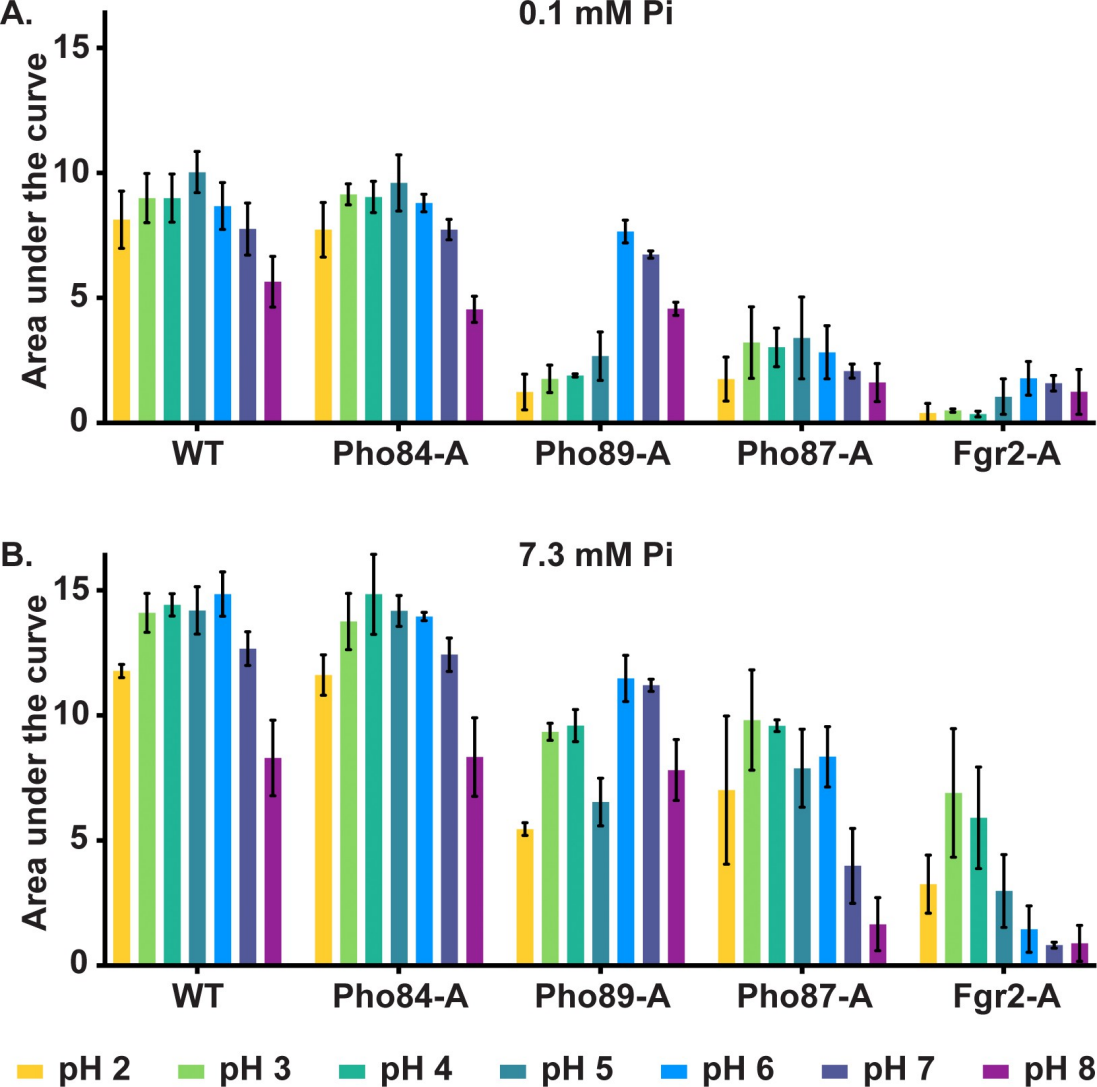
Pi transporters differed in their optimal pH and Pi concentration range while Pho84 was most active overall. Cells expressing the indicated transporter alone among the 4 Pi transporters were inoculated to an OD_600_ of 0.1 into SC medium buffered to the indicated pH and grown in a plate reader at 30°C for 20 h. OD_600_ was measured every 15 min and the area under the growth curve was calculated in Graphpad Prism; means of 3 biological replicates are depicted in histograms. See Fig C in S1 Text for all growth curves and Fig D in S1 Text for statistical analysis. Error bars represent SD of 3 biological replicates. **A.** SC containing 0.1 mM KH_2_PO4. **B.** SC containing 7.3 mM KH_2_PO4. Strains are WT (JKC915); Pho84-A: *pho87-/- pho89-/- fgr2-/- PHO84+/+* (JKC2788); Pho87-A: *pho84-/- pho89-/- fgr2-/- PHO87+/+* (JKC2777); Pho89-A: *pho84-/- pho87-/- fgr2-/- PHO89+/+* (JKC2783); Fgr2-A: *pho84-/- pho87-/- pho89-/- FGR2+/+* (JKC2758). No significant difference was observed between Pho84-A and WT cells under any tested conditions (by two-tailed Student’s t-test).

We defined the pH range at which each triple mutant retaining a single predicted Pi transporter was able to grow in SC medium with low or high Pi. Growth was assayed by optical density and the area under each growth curve (AUC) was depicted as a histogram (Fig 4 and Figs C and D in S1 Text). Substantial growth was defined as growth ≥ AUC 5. Overall, we found that growth of Pho84-Alone (*pho89-/- pho87-/- fgr2-/-*) cells resembled that of WT (Fig 4), with growth optima between pH 2 and pH 7 in both high and low Pi conditions.

The role of Pho89 in growth was dependent on pH and Pi availability (Fig 4). In low Pi, Pho89-Alone cells grew equivalently to WT at pH 6 and above. In high Pi, Pho89 supported intermediate levels of growth at more acidic pH and growth equivalent to WT at pH 6 and above. Pho89 could hence be described as a putative high-affinity Pi transporter in neutral and alkaline conditions and a low-affinity transporter in acidic conditions. *C*. *albicans*, like *S*. *cerevisiae*, therefore has two high-affinity Pi transporters, Pho84 and Pho89, with the former having a broad pH activity range including in alkaline conditions, and the latter active at pH ≥6.

Pho87 and Fgr2 were unable to support substantial growth in low ambient Pi and therefore are low-affinity Pi transporters. Both supported growth only at acidic pH in high Pi. Pho87-Alone cells grew to an AUC ≥5 only between pH 2 and 6, and even at their optimal conditions supported only ~70% of the WT growth (Fig 4 and Fig D in S1 Text). Fgr2-Alone cells showed the weakest growth with similar optima to Pho87-Alone cells, growing to an AUC ≥5 only at pH 3 and 4 (Fig 4 and Fig D in S1 Text). *C*. *albicans* therefore has two low-affinity Pi transporters, Pho87 and Fgr2, with the latter, a Pho84 homolog, showing a narrow, acidic pH optimum.

Hyphal formation of triple Pi transporter mutants largely reflected the growth-sustaining properties of the transporters. Q- mutants lacking all four Pi transporters failed to form hyphae under any conditions tested (Fig 2). In contrast, Pho84-Alone cells encoding only Pho84 formed robust hyphae across all conditions (Fig 2), consistent with the strong hyphal defect of *pho84-/-* mutants. Pho89-Alone cells had severe hyphal growth defects resembling those of *pho84-/-* mutants. Filamentation of the Pho87-Alone and Fgr2-Alone cells resembled the Q- mutants (Fig 2). These mutants did not grow sufficiently to form hyphae on RPMI buffered to pH 7 (Fig 2C). Collectively, these findings are consistent with a concept that hyphal growth requires Pi uptake.

### Pho84 showed the most active Pi uptake under all tested pH conditions

**Fig 5:**
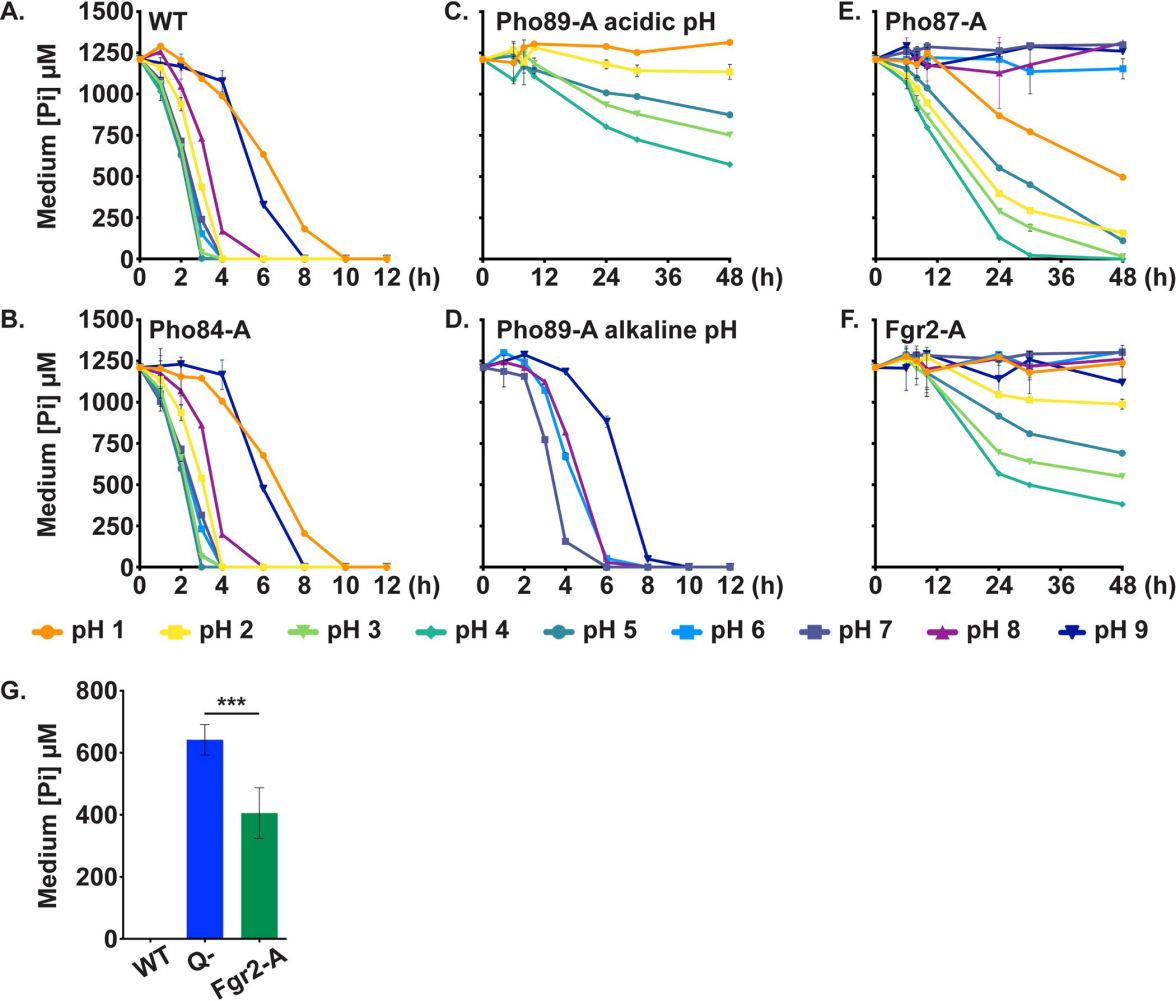
Pi uptake of cells expressing single Pi transporters reflected their growth optima. Cells with indicated genotypes were inoculated into SC without Pi (buffered to pH 1–9) at OD_600_ 2. After 30 minutes, KH_2_PO_4_ was added to a final concentration of 1 mM, and the extracellular concentration of phosphate was measured in 2 technical replicates at indicated time points. Error bars SD. Representative of three biological replicates. **A.** WT (JKC915). **B.** Pho84-A: *pho87-/- pho89-/- fgr2-/- PHO84+/+* (JKC2788). **C.** Pho89-A in pH 1–5; **D.** Pho89-A in pH 6–9: *pho84-/- pho87-/- fgr2-/- PHO89+/+* (JKC2783). **E.** Pho87-A: *pho84-/- pho89-/- fgr2-/- PHO87+/+* (JKC2777). **F.** Fgr2-A: *pho84-/- pho87-/- pho89-/- FGR2+/+* (JKC2758). **G.** Q- cells (*pho84-/- pho87-/- pho89-/- fgr2-/-;* JKC2830) took up significantly less Pi at pH 4 than Fgr2-A cells at 30 h of incubation. Histograms depict average and SD of 3 biological replicates, *p* = 0.0001 (two-tailed Student’s t-test).

In order to quantify the Pi transport capacity of each transporter, we performed Pi uptake experiments with the triple mutants that each retained a single predicted transporter, by measuring Pi concentrations remaining in culture medium over a time course. The experiments were performed at a defined cell density, OD_600_ of 2, so that in the first 4–6 hours the uptake per cell was comparable across strains, though at later time points differential cell growth and division may have influenced uptake experiments. WT and Pho84-Alone cells removed Pi from the medium rapidly and with almost identical kinetics (Fig 5A and 5B). Pho89-Alone cells efficiently transported Pi at a narrow range of pH 6–8, but their uptake dramatically slowed at pH 5 and below (Fig 5C and 5D). These results support a predominant role of Pho84 as the major Pi importer in *C*. *albicans*, while Pho89 makes a substantial contribution around neutral pH.

Low-affinity Pi transporters Pho87 and Fgr2 showed slow Pi uptake under these conditions. Uptake of Pi by Pho87-Alone cells was sluggish at pH 2–5 and almost undetectable at pH 6–9 (Fig 5E). Despite known strong induction of *FGR2* in low Pi conditions by Pho4, the transcriptional regulator of the PHO regulon [64], Pi uptake by Fgr2-Alone cells was weak across the pH levels tested and these cells were unable to fully deplete Pi from the medium at their pH 4 optimum (Fig 5F). Both Pho87-Alone and Fgr2-Alone cells removed Pi from the medium most rapidly at pH 4. Still, Pi uptake by Fgr2-Alone cells was significantly higher than that of Q- cells at 30 hours (*p* = 0.0001 by two-tailed t-test, Fig 5G). These data support a role for Pho87 and Fgr2 as Pi importers albeit with poor kinetics.

To identify any growth defects unrelated to Pi limitation in mutants containing a single Pi transporter, we compared growth of triple mutants to WT in liquid YPD and Pi replete SC media. YPD contains organic as well as inorganic phosphate sources, and SC contains high Pi concentrations (7.3 mM) and has an acidic pH of 4–5 that favors activity of most Pi transporters. No triple mutants exhibited a growth defect in YPD medium. In SC medium, Pho84-Alone grew as well as WT while Pho87-Alone, Pho89-Alone and Fgr2-Alone cells grew more slowly (Fig E in S1 Text), consistent with our previous results (Fig 1 for SC medium with 7.3 mM Pi at pH 5). These findings indicate that growth defects in these mutants correspond to a lack of Pi and not a nonspecific fitness loss. Single- and triple mutant phenotypes taken together highlight that Pho84 plays a central role in Pi uptake and in cellular functions ranging from oxidative stress management to hyphal growth (Table 1).

**Table 1:** Pi transporter characteristics.

	Pho84	Pho89	Pho87	Fgr2
**Chromosomal location**	Chr1	Chr4	Chr1	Chr7
**Pi affinity**	high	high	low	low
**Optimal pH range**	pH 2–8	pH 6–8	pH 2–6	pH 3–4
**Role in hyphal growth**	+++	+	-	-
**Role in Target of Rapamycin Complex1 signaling**	Yes	No	No	No
**Role in oxidative stress response**	Yes	No	No	No

### Glycerophosphocholine transporters provided a minor Pi import function

**Fig 6:**
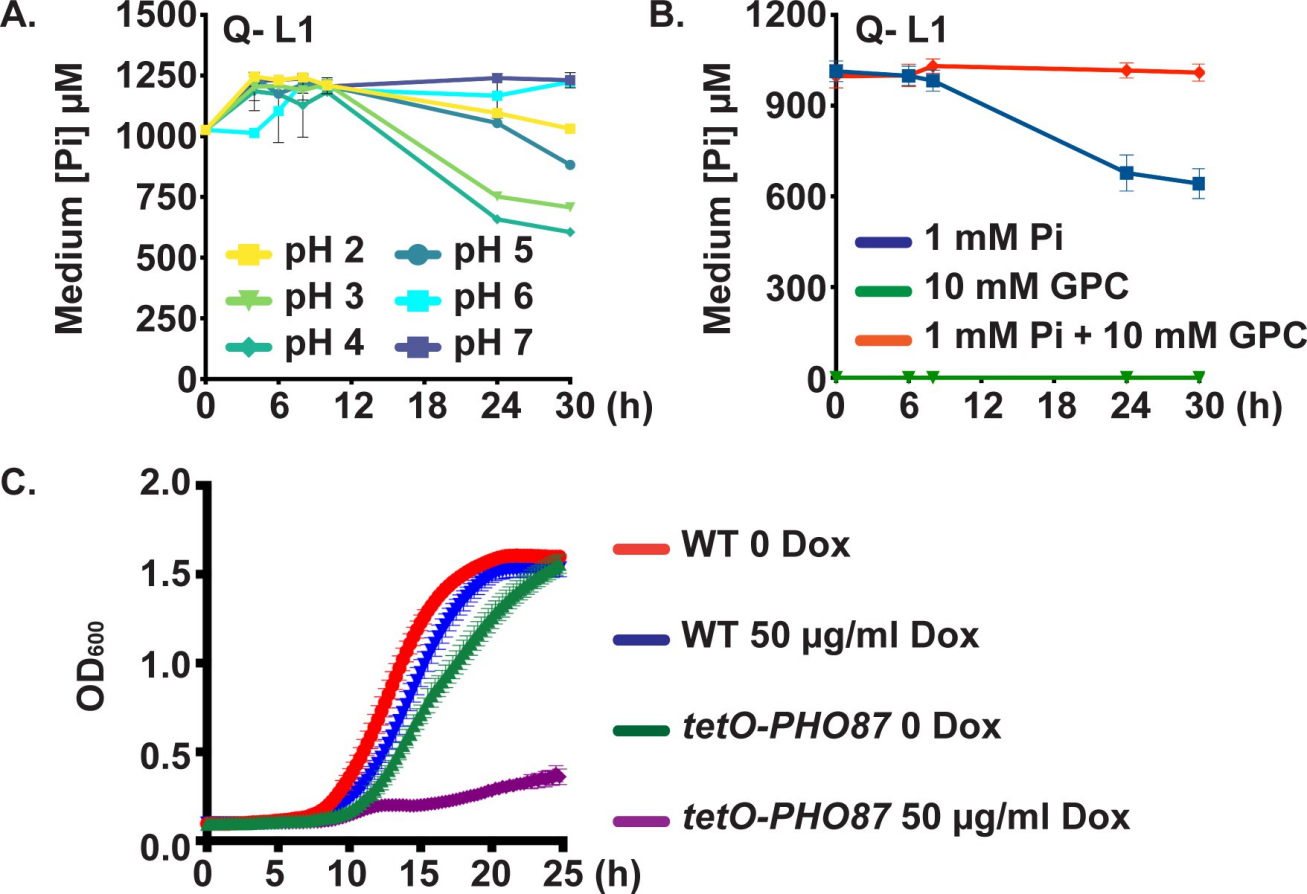
Cells lacking 4 Pi transporters showed residual Pi uptake ability that was outcompeted by glycerophosphocholine. **A.** Pi uptake experiments performed as in Fig 5 showed that in Q- cells (*pho84-/- pho87-/- pho89-/- fgr2-/-*, JKC2830) residual Pi uptake occurred and was most efficient at pH 4. **B.** Tenfold excess glycerophosphocholine (GPC) inhibited Pi uptake in Q- cells at pH 4. As in Fig 5, Q- cells (JKC2830) were inoculated into SC 0 Pi with 10 mM GPC; after 30 minutes, KH_2_PO_4_ was added to a final concentration of 1 mM; Pi concentration in the medium was measured with 2 technical replicates at each time point. Graph shows mean of 3 biological replicates. Error bars SD. **C.** Cells in which *PHO87* is expressed from repressible *tetO* while the other 3 Pi transporters and *GIT2-4* are deleted (*tetO-PHO87/pho87 pho84-/- pho89-/- fgr2-/- git2-4-/-*, JKC2969), grew well in the absence of doxycycline but grew minimally during *tetO* repression in 50 μg/ml doxycycline. WT (JKC915) and *tetO-PHO87* (JKC2969) were starved for Pi in SC 0 Pi in the presence of 50 μg/ml doxycycline for 48 h. The medium and doxycyline were replaced at 24 h. Cells were then inoculated at OD 0.1 into SC medium (7.3 mM Pi), buffered to pH 3, without and with 50 μg/ml Doxycycline. OD_600_ was recorded every 15 min. Error bars SD of 3 technical replicates. Representative of 3 biological replicates.

To test whether *C*. *albicans* expresses additional Pi transporters that were not detected by our homology searches, we next examined Q- cells for their ability to import Pi. At 30 hours, Q- mutants took up ~40% of the Pi at pH 4 with efficiency being further reduced at pH 2, 5, 6 and 7 (Fig 6A). These results demonstrated that another low-capacity Pi transporting activity existed at low pH and high Pi concentrations.

*C*. *albicans GIT3* and *GIT4* are distant *PHO84* homologs whose products are known to import glycerophosphocholine (GPC), a phospholipid degradation product that can serve as an organic source of phosphorus [66]. Once in the cytosol, GPC is metabolized to glycerol, choline and phosphate under Pi limiting conditions [66]. The GPC transporters Git3 and Git4 share 23% amino acid identity with Pho84 (41% and 39% similarity, respectively, Fig A in S1 Text). To test whether Git3 and Git4 might contribute to the residual Pi transport in Q- cells, we competed Pi uptake by Git3 and Git4 with excess GPC in the medium. Addition of a 10-fold excess of GPC completely eliminated Pi uptake by Q- cells (Fig 6B). These findings support a role for Git3/4 in Pi import at high ambient Pi and acidic pH.

We asked whether *C*. *albicans* has another modality to import Pi from its surroundings, in addition to the 2 high-affinity Pi transporters Pho84 and Pho89, the 2 low-affinity Pi transporters Pho87 and Fgr2, and the GPC transporters Git3 and Git4. We engineered a septuple mutant strain “*tetO-PHO87”* that lacked three Pi transporter homologs (*pho84-/- pho89-/- fgr2-/-*), GPC transporters that occupy adjacent loci on chromosome 5 (*git2-/- git3-/- git4-/-*), and had a single tetracycline-repressible allele of *PHO87* (*pho87-/tetO-PHO87)*. Construction of this strain was independent of that of Q- strains and is described in detail in Methods and S2 Table. Mutants were maintained without doxycycline to retain maximal expression of *PHO87*. A role for Git2 in Pi import is currently not known; *GIT2* was deleted alongside the other two transporters in their initial characterization and it was included in this construct to permit comparisons with mutants described in Bishop et al. [66].

We reasoned that if a Pi-transporting activity remained in these cells, they would grow in Pi as their only source of phosphorus, both in the absence and presence of doxycycline, i.e., during induction and repression of *PHO87*. On the other hand, if we had mutated all transporters capable of importing Pi, doxycycline exposure in SC media, devoid of organic phosphate sources, would repress growth once internal Pi stores were depleted. We observed the latter result for the *tetO-PHO87* septuple mutants (*pho87-/tetO-PHO87 pho84-/- pho89-/- fgr2-/- git2-/- git3-/- git4-/-*) (Fig 6C). In contrast at pH 3, an optimal pH for Pho87 activity, these mutants grew robustly in media without doxycycline, YPD or normal SC (which contains high Pi, 7.3 mM) though they had a slight growth defect compared to WT (Fig 6C). Thus, the reduced growth of these mutants is largely attributable to Pi starvation and not a consequence of a nonspecific fitness loss (Fig F in S1 Text). We concluded that minimal Pi import activity remains when *PHO84*, *PHO89*, *PHO87*, *FGR2* and *GIT2-4* have been genetically eliminated.

### In vitro evolution during Pi scarcity restored fitness to different degrees in two distinct quadruple Pi transporter mutant lineages

**Fig 7:**
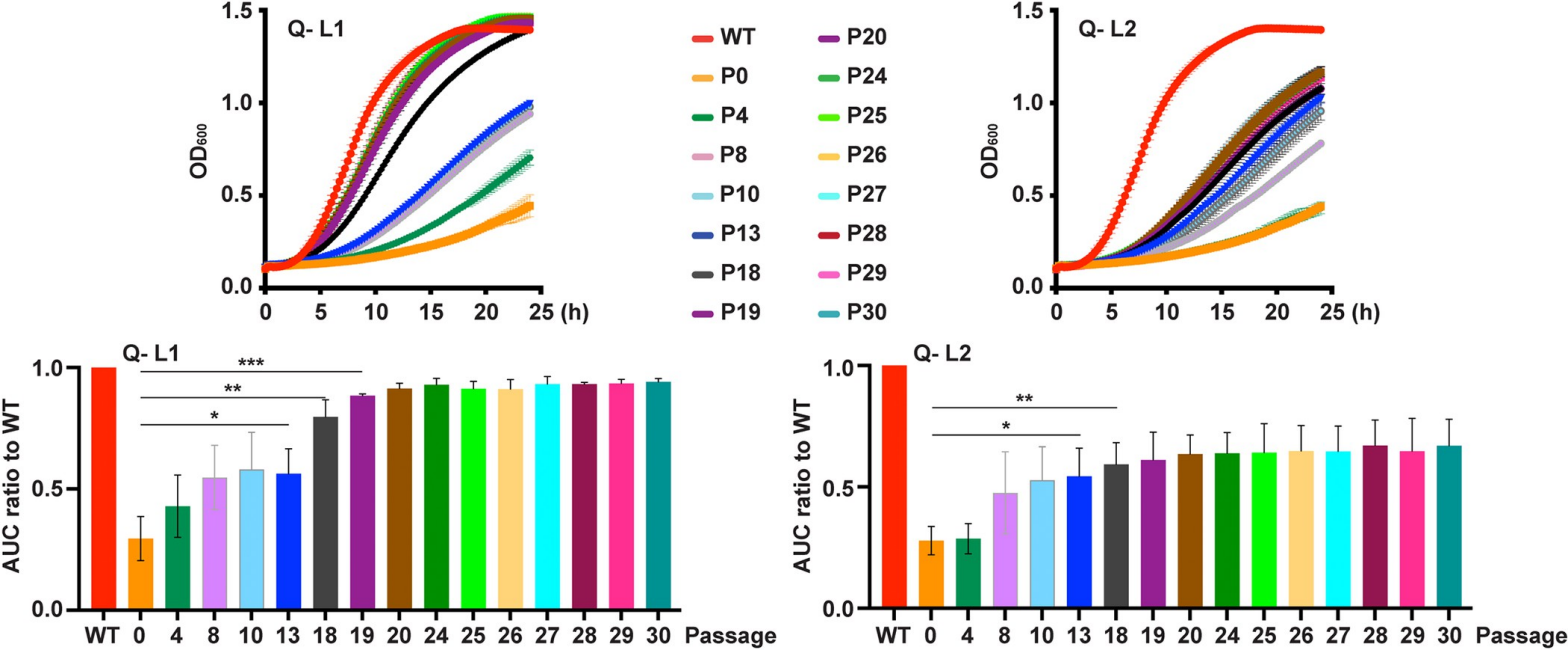
Evolution of 2 Q- lineages during Pi scarcity proceeded along distinct trajectories. Q- L1 and -L2 cells and selected passages from in vitro evolution were grown in SC 0.4 mM Pi (*pho84-/- pho87-/- pho89-/- fgr2-/-*, JKC2830, Q- L1 and JKC2860, Q- L2). Representative growth curves and corresponding area under the curve (AUC) shown for selected passages. P0 denotes the Q- isolate before passaging; all experiments after P0 were performed with populations, not with clones derived from single colonies. Growth curves are representative of 3 biological replicates, except for passages 8 and 10, which are representative of 2 biological replicates. Error bars SD of 3 technical replicates. For statistical significance: ns, *p* > 0.05; *, 0.01 < *p* ≤ 0.05; **, 0.001 < *p* ≤ 0.01; ***, 0.0001 < *p* ≤ 0.001 by two-tailed Student’s t-test.

We reasoned that in vitro evolution of *C*. *albicans* Q- mutants lacking all four Pi transporter homologs (*pho84-/- pho89-/- pho87-/- fgr2-/-*) could reveal adaptive mechanisms that facilitate growth during Pi scarcity. Our previous work showed that *pho84*-/- mutants’ cell wall contained less phosphomannan and had a thinner outer layer [27], suggesting that modifying cell wall structures while reserving scarce Pi for essential processes like nucleic acid biosynthesis could sustain Pi-starved cells. We propagated two lineages of the Q- mutants through 30 serial passages every other day in liquid SC medium with a moderate concentration of 0.4 mM Pi. To reduce genetic bottlenecks, a large number of cells (~3.5x10^6^ cells in 10 μl of saturated culture, as opposed e.g. to 8x10^3^ in [67]) were reinoculated into 10 ml of fresh medium. The population at each passage was saved for DNA extraction and as a glycerol stock. During passaging, the growth rates of both Q- lineages increased substantially and in distinct increments. Growth rates of the Q- lineages ultimately plateaued before the end of the experiment. However, fitness recovery in the two lineages during passaging differed significantly. Lineage 1 (Q- L1, evolving from JKC2830), recovered fitness more rapidly and attained a growth rate similar to the WT by passage 24 (Fig 7). In contrast, growth rates of populations derived from lineage 2 (Q- L2, evolving from JKC2860) remained lower than WT throughout the experiment, plateauing at passage 20 (Fig 7). Fitness loss of Q- strains and fitness recovery of their descendant passaged populations was specific to Pi scarcity. In YPD, both Q- strains and their 30^th^ passage descendant populations grew robustly (Fig F in S1 Text). These findings indicated that *C*. *albicans* cells lacking Pi transporters could recover fitness during prolonged Pi deprivation, and that this adaption did not lead to fitness loss when the selective pressure was relieved.

### Specific stress phenotypes of Q- population passages did not consistently correspond to their fitness in Pi scarcity

**Fig 8:**
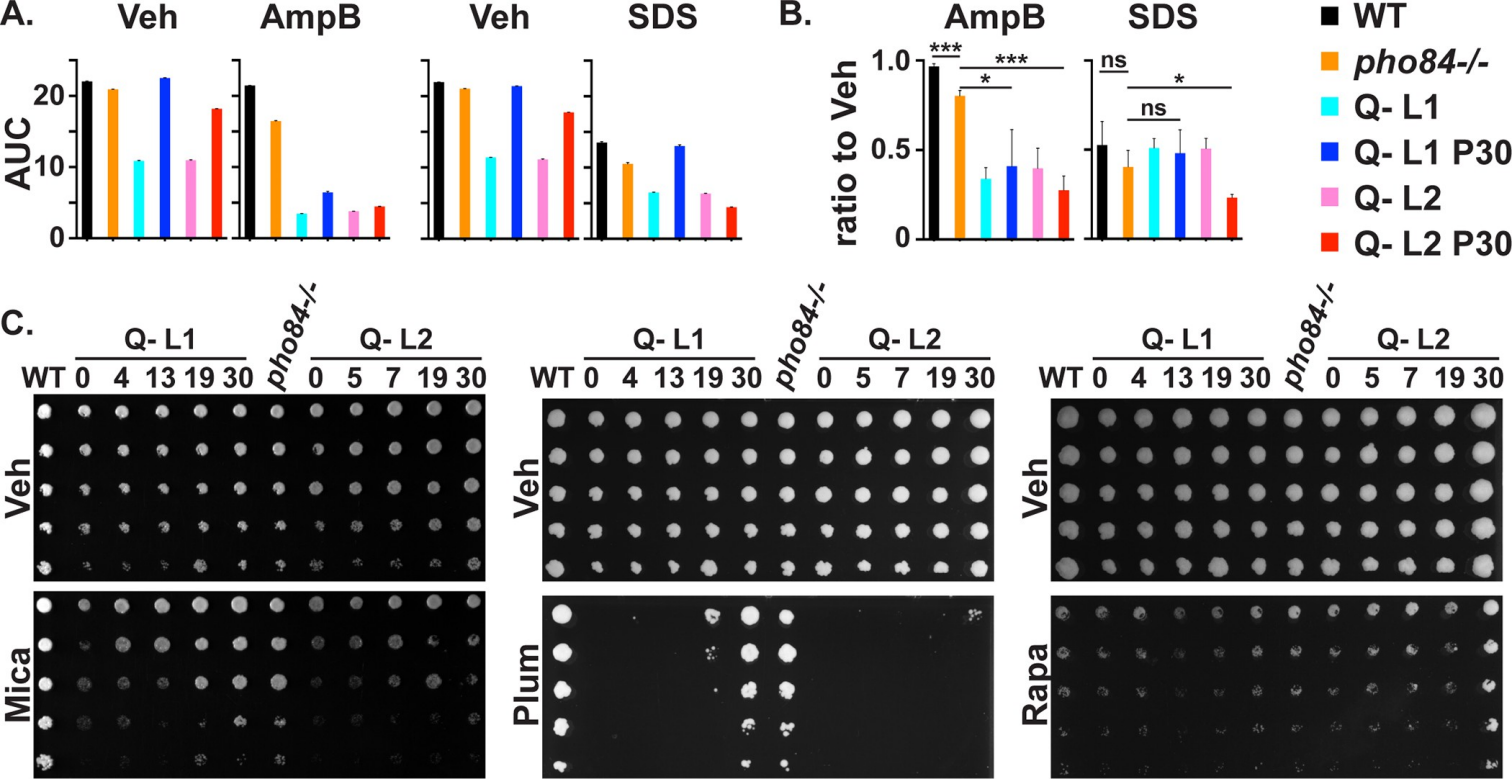
Two evolving lineages of Q- cells showed distinct stressor endurance. **A.** Growth area under the curve (AUC) of cells grown in SC 1 mM Pi containing Vehicle (Veh, H_2_O), 0.35 μg/ml Amphotericin B (AmpB) or 0.005% SDS. Representative of 3 biological replicates; error bar SD of 3 technical replicates. **B.** AmpB and SDS growth AUC from panel A normalized to each strain’s vehicle control. WT (JKC915); Q- L1 (JKC2830); Q- L1 P30 (JKC2830 passage 30); Q- L2 (JKC2860); Q- L2 P30 (JKC2860 passage 30). Error bar SD of 3 biological replicates. For statistical significance: ns, *p* > 0.05; *, 0.01 < *p* ≤ 0.05; ***, 0.0001 < *p* ≤ 0.001 by two-tailed Student’s t-test. **C.** Threefold dilutions of cells of indicated genotypes, starting at OD_600_ 0.5, were spotted (top to bottom) onto SC medium containing Vehicle (Veh, H_2_O) or 10 ng/ml micafungin (Mica), 15 μM plumbagin (Plum), 50 ng/ml rapamycin (Rapa), and grown at 30°C for 1 d (Mica), 2 d (Plum), 4 d (Rapa), respectively. Strains are WT (JKC915); Q- L1 (JKC2830) passage 0, 4, 13, 19, 30 and Q- L2 (JKC2860) passages 0, 5, 7, 19, 30; *pho84-/-* (JKC1450). JKC2830 and JKC2860 genotypes are *pho84-/- pho87-/- pho89-/- fgr2-/-*. Representative of 3 biological replicates.

As we previously found hypersensitivity of *pho84*-/- mutants to rapamycin as well as oxidative-, cell wall- and membrane stress, induced through plumbagin, micafungin and amphotericin exposure respectively [8,27], we investigated these responses in the Q- mutants and their evolved lineages. WT, both Q- strains and their passage 30 descendant populations were grown in liquid medium; these strains and in addition, populations from intermediate passages from the evolution experiment were spotted as serial dilutions onto solid medium in the absence or presence of stressors. Q- mutants were hypersensitive to membrane stress induced by amphotericin and SDS and to cell wall stress induced by micafungin; they showed rapamycin sensitivity corresponding to their growth defects in vehicle (Fig 8).

In contrast, the responses of evolved, late-passage Q- L1 and Q- L2 populations were distinct for each stressor. Q- L1 but not Q- L2 populations regained growth rates in amphotericin almost to WT levels by passage 30 (Fig 8A and 8B). The Q- L2 passage 30 population had increased sensitivity to SDS, compared with its ancestral Q- L2 strain while its Q- L1 counterpart regained the ability to grow in the presence of SDS almost to the level of the WT (Fig 8A and 8B). In the presence of micafungin, growth of selected passages reflected their fitness in Pi-limited medium (Fig 8C). However, Q- L1 but not Q- L2 passaged populations regained growth in plumbagin while conversely, the Q- L2 population evolved frank resistance to rapamycin by passage 30 (Fig 8C). Rapamycin resistance was the only phenotype in which fitness of an evolved population exceeded fitness of the *pho84-/-* mutant. Like the different fitness levels in Pi scarcity reached by the end of the experiment, the distinct stress phenotypes of Pi scarcity-adapted populations suggest their evolutionary trajectories had diverged.

### Acquisition and loss of aneuploidies accompanied adaptation of evolving Q- lineages to Pi scarcity

To uncover the underlying genomic changes associated with improved growth of evolving Q- strains, we performed whole genome sequencing of the WT ancestral to the Pi transporter mutants (JKC915), as well as cell populations of selected passages from both lineages, Q- L1 and Q- L2. Populations that bounded significant fitness gains were chosen for sequencing, generating genomic snapshots of the evolved population for passages 4, 13, 18, 19, and 30 of Q- L1, and passages 5, 7, 19, 24, and 30 for Q- L2.

**Fig 9:**
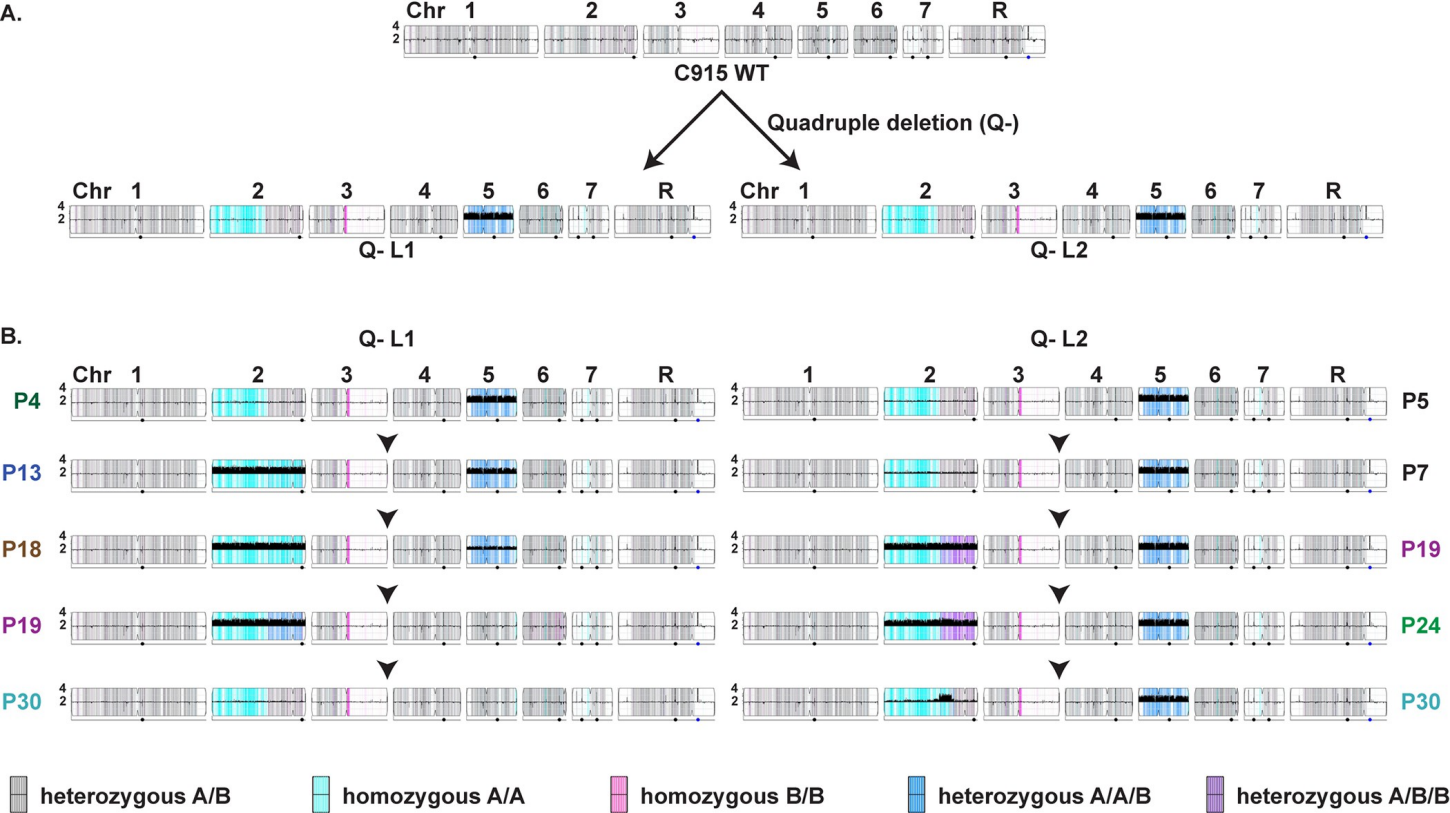
Whole genome sequencing of selected passages of 2 evolving Q- derived populations showed distinct trajectories of acquisition and resolution of aneuploidies and loss of heterozygosity. **A**. YMAP [85] depictions of WGS results of 2 distinct Q- isolates showing Chr5 trisomy and loss of heterozygosity of Chr2 and Chr3, compared with their progenitor JKC915, the WT used in this study. Chromosomes are represented lengthwise on the horizontal axis, with centromeres denoted by the pinched position. The vertical height of the solid black bars along the horizontal axis indicates copy number in 10 kilobase bins (flat black line = 2N). Gray background shades indicate local SNP densities, such that darker gray shades correspond to higher and lighter shades to lower SNP densities. Gray, cyan, and magenta colors represent heterozygous, homozygous homolog A, and homozygous homolog B regions respectively. Blue indicates A/A/B allelic balance, and purple indicates A/B/B allelic balance. Black circles under a chromosome indicate the location of the major repeat sequence (MRS) loci, and the blue circle under ChrR represents the ribosomal DNA cluster. B. YMAP depictions of WGS results of populations evolving from JKC2830, Q- L1 and JKC2860, Q- L2 (both *pho84-/- pho87-/- pho89-/- fgr2-/-*). YMAP symbols as in A.

Major chromosomal rearrangements occurred during construction of the Q- mutants and during their passaging. The WT strain used to construct the Q- mutant lineages was diploid with no evidence of segmental copy number variations or large loss of heterozygosity (LOH) tracts. Of note, the genetic background used in our and most other *C*. *albicans* genetic experiments, SC5314 [68], is largely heterozygous across its genome and the Candida Genome Database [69] denotes the distinct alleles of its diploid genome as A or B. The genomes of both Q- strains were trisomic for chromosome 5 (Chr5, Fig 9A), which includes *GIT2-4*. This aneuploidy was accompanied by LOH tracts on the left arm of Chr2 (Chr2L) and Chr3R, neither of which include loci disrupted in the Q- mutant. LOH of Chr2 resulted in homozygosis of the A allele for >50% of the chromosome, and LOH of Chr3 produced a short tract of homozygosity for B alleles (Fig 9A).

Both evolving lineages acquired similar large-scale genomic changes during adaptation to Pi scarcity. Each lineage independently acquired a Chr2 trisomy, first seen in passage 13 and passage 19 for Q- L1 and Q- L2, respectively (Fig 9B). The Chr2 trisomy was subsequently largely or partially lost in Q- L1 and Q- L2, respectively, as populations accumulated fitness-enhancing small-scale mutations. The Chr5 trisomy was also lost during passaging of Q- L1, likely due to fitness defects associated with aneuploidy (Fig 9B).

Persistence of Q- L2’s ~300 kb segmental aneuploidy on the left arm of Chr2 (Fig 9B) by the 30^th^ passage suggests potential adaptive contributions of the encoded loci. This amplified region of Chr2 ranged from nt 1,316,396 to 1,617,025 and encompassed 147 open reading frames. Manual annotation of these genes (S1 Table) identified *GIT1*, which encodes a glycerophosphoinositol permease [70] with 22% amino acid sequence identity to Pho84. There is experimental evidence against a role of Git1 in Pi transport in *C*. *albicans* [70] but its role in phosphorus homeostasis might be indirect, i.e. by facilitating glycerophosphoinositol uptake as Pi starved cells induce plasma membrane remodeling [71]. Other potentially relevant genes on this chromosomal segment are orf19.2268, which encodes a homolog of the *S*. *cerevisiae* serine/threonine protein kinase Rck2 that is induced by the oxidative stress-responsive Hog1 MAP kinase and whose mutants are rapamycin hypersensitive [72], and orf19.2290, which encodes the Tor1 kinase (S1 Table).

Quantifying the small-scale variants that accumulated across selected passages, we found that the Q- L1 lineage acquired overall more variants than the L2 lineage (Table 2). Variant calling was used to identify large-effect variants [73] like indels in coding sequences, that were detectable in both lineages and that were present in one or both lineages at the last passage, as we reasoned that such mutations might be good prospects for contributors to increased fitness during Pi scarcity. *PTP2* is a possible candidate regulator in whose coding sequence a 2-nucleotide deletion occurred in both lineages; this frame shift-inducing deletion was fixed in Q- L1 by P19 but not in Q- L2. *PTP2* encodes a predicted tyrosine phosphatase that contributes to maintenance of hyphal growth after hyphal induction by Tor1 or serum exposure [74]. Another possible candidate gene differentially mutated in the 2 lineages per SnpEff analysis is *FET99*, which encodes a multicopper oxidase. In this gene, a 2-nucleotide frameshift-inducing deletion occurred by P5 in Q- L2 and remained fixed for all remaining passages. In Q- L1, this deletion appeared intermittently but resolved by P30, presumably as clones that still contained wild type *FET99* took over the population. Fet99 is a homolog of *S*. *cerevisiae* Fet3 and may have a role in reductive iron uptake, as its transcription is strongly regulated by iron provision versus -depletion [75]. While these findings are only the beginning of our in-depth genetic and physiologic analysis, they indicate novel lines of investigation to illuminate the adaptations available to *C*. *albicans* as it confronts a broad spectrum of Pi availability as a commensal and an invasive pathogen.

**Table 2:** Accumulation of genomic variants over successive passages of evolving Q- populations.

**Q- L1 (JKC2830)**					
**Passage**	**P0 to P4**	**P4 to P13**	**P13 to P18**	**P18 to P19**	**P19 to P30**
**New variants**	269	254	279	367	163
**Number of passages**	4	9	5	1	11
**Average # variants per passage**	67.25	28.22	55.8	367	14.82
**Q- L2 (JKC2860)**					
**Passage**	**P0 to P5**	**P5 to P7**	**P7 to P19**	**P19 to P24**	**P24 to P30**
**New variants**	262	281	262	414	199
**Number of passages**	5	2	12	5	6
**Average # variants per passage**	52.4	140.5	21.83	82.8	33.17

## Discussion

In this work, we characterized 4 predicted *C*. *albicans* Pi transporters, Pho84, Pho89, Pho87 and Fgr2, identified by sequence homology, and determined their contributions to Pi acquisition. In brief, among the high-affinity Pi transporters, Pho84 was the most important for growth, filamentation, stress responses, and induction of TORC1 signaling and had the broadest pH range of Pi uptake capacity, while Pho89 was specialized for uptake in neutral and alkaline pH (Table 1). Among the low-affinity Pi transporters, both of which were only active at acidic pH, Pho87 was more efficient and had a broader pH range while Fgr2 functioned only between pH 3 and 5 (Figs 4 and 5). In contrast to *S*. *cerevisiae*, *C*. *albicans* low-affinity Pi transporters are not paralogs; rather, the less efficient one, Fgr2, is a distant Pho84 homolog. The minor role for Fgr2 in *C*. *albicans* Pi import stands in contrast to *C*. *neoformans* where both *PHO84* homologs (*PHO84* and *PHO840*) make significant contributions to Pi transport [76]. In the absence of all specific Pi transporters, glycerophosphocholine transporters were able to provide residual Pi import to sustain growth. Cells lacking all Pi transporters were able to regain fitness during sequential passaging in limited Pi. Their fitness recovery plateaued at distinct levels for 2 populations in accordance with previously described declining adaptability [77,78], while engendering distinct responses to some Pi-relevant stressors. During the evolution experiment, similar large-scale genomic changes were sequentially acquired and partially or completely lost in the 2 independently evolving populations. Retention of a segmental amplification within Chr2 in the less fit lineage suggests that genes in this trisomic region may contribute to fitness in limited Pi. Candidate genes residing on this segment that could contribute to fitness recovery, *GIT1*, *PCK2* and *TOR1*, await further genetic confirmation.

The severe growth defect of Q- cells argues against the presence of other specific Pi transporters. Q- cells removed a small fraction of the Pi present in their medium. The glycerophosphocholine transporters Git3 and 4 provided minor Pi import activity. The contribution of Git3/4 was most evident in the growth difference between a Q- mutant and the septuple mutant when grown in the presence of doxycycline to repress *PHO87* (Fig 6C). The ability of GPC provision to completely outcompete measurable Pi uptake in Q- cells (Fig 6B) also argues against other cell surface transporters beside Git3/4 playing a measurable role in Pi uptake under our experimental conditions.

pH sensitivity is a key feature of each transporter. Like WT, Pho84-Alone, Pho87-Alone, and Fgr2-Alone cells grew optimally between pH 3 and 6. Only cells expressing *PHO89* alone showed a growth optimum between pH 6 and 8. The pH of the oral and pharyngeal mucosa as well as most of the gastrointestinal tract colonized by *C*. *albicans* is broadly neutral or alkaline, though mucosal microenvironments may be acidic due to bacterial metabolites. During acute invasive disease, *C*. *albicans* finds itself in mildly alkaline blood and tissue environments between pH 7.35 and 7.45. Pi acquisition systems of *C*. *albicans* are therefore not well adapted to host environments encountered during invasive disease. The reduced Pi transport activity at alkaline pH of the bloodstream might explain why the PHO regulon is induced during systemic disease, reflecting “alkaline pH-simulated nutrient deprivation” [79], despite the presence of abundant Pi and organic phosphate compounds like GPC. Pi import is also critical for proliferation of other human fungal pathogens [31,80,81] and unicellular parasites [82] each of which must contend with neutral to alkaline conditions in host deep organs.

Loss of *PHO84* but not of the other Pi transporters had a substantial effect on TORC1 activity (Fig 3) and oxidative stress endurance (Fig B in S1 Text). These experiments cannot distinguish between specific activities of Pho84 in these cellular functions, versus the predominant role of Pho84 in providing Pi to the cell. We found in another context that TORC1 activity depends on availability of Pi but not on the presence of Pho84, while endurance of peroxide stress may require an activity specifically of Pho84 [83]. *C*. *albicans PHO84* transcription is co-regulated by TORC1 in addition to Pho4 [8], but how these systems interact and modulate each others’ outputs remains unknown.

Hyphal growth defects mirrored the severity of Pi transport deficiency among the constructed mutants. Among the Pi transporters assayed in defined mutants, only Pho84-Alone cells produced robust hyphae on all 3 media examined (Fig 2), while Pho87-Alone cells produced some hyphae on RPMI pH 5, and Pho89-Alone cells had sparse, short hyphae on RPMI pH 7 (Fig 2). The role of Pi uptake in filamentation might be indirect, through activation of signaling systems like TORC1 required for hyphal morphogenesis [84]. Alternatively, hyphal cells with their larger surface area than yeast cells, must consume larger amounts of phosphoric metabolites like nucleotide sugars for cell wall construction. Lack of phosphorus-containing intermediates might induce regulatory steps to minimize cell wall surface area and preserve phosphorus for other vital functions and therefore to inhibit hyphal morphogenesis.

To probe *C*. *albicans’* options for adaptation to Pi scarcity, we performed an in vitro evolution experiment with Q- strains in which we had deleted the known Pi transporters. These strains had acquired, at an unknown point during their construction, a trisomy of Chr5 where the genes encoding organic phosphate transporters Git3/4 reside. During the evolution experiment, a further large-scale genomic alteration appeared in both lineages: triploidy of Chr2 with simultaneous LOH reducing all 3 alleles to homozygous AAA in a long segment on the left arm of the chromosome (Fig 9) (cyan-colored segment of Chr2 in both lineages). (In the YMAP graphic depiction of WGS results [85], the 2 distinct alleles of each chromosomal region are represented by colors: gray represents heterozygous regions, cyan indicates homozygous homolog A, and magenta indicates homozygous homolog B regions.) Aneuploidy and LOH are known to occur in *C*. *albicans* in persistent stress conditions [50–52,86,87], reviewed in [88]. Ploidy increases that enhance fitness during exposure to a specific stress, occur more rapidly than accumulation of advantageous point mutations, because chromosome missegregation occurs once every 5 × 10^5^ cell divisions (in *S*. *cerevisia*e) [89] while substitution of any particular base pair is estimated to occur once every 1.2 × 10^10^ cell divisions in *C*. *albicans* [90] and every 1.67 × 10^10^ cell divisions in *S*. *cerevisiae* [91]. Populations under strong selection are therefore more likely to initially become enriched for aneuploid mutants than for cells containing constellations of advantageous point mutations [92]. Large-scale copy number variants like trisomies however incur fitness costs due to increased transcription and translation of a multitude of genes that result in excess protein production and protein complex stoichiometry imbalances [89,93,94]. These fitness costs predominate when the selective pressure of the stress relents [50,94]. In addition to environmental changes that diminish stress intensity, small-scale genomic changes (like single nucleotide mutations and small insertions or deletions) that promote adaptation to the specific stressor can relieve selective pressure and favor loss of a trisomic chromosome. For example, a gain-of-function point mutation of a transcriptional regulator [50,95], may alleviate a specific stressors’ selective pressure to the point that trisomies resolve back to diploid, as we observed in the Q- L1 but not the Q- L2 lineage.

Evolving quadruple Pi transporter mutants similarly gained trisomies in both independent populations, highlighting the strong selection experienced by these cells. Fitness and stress resistance phenotypes of each passage’s population hence were due to a combination of large- (structural) and small scale genomic changes. Loss of trisomies during passaging may have been enabled by accumulating fitness-enhancing small-scale variants (Table 2). The constellation of mutations that permitted these adaptive solutions remain to be identified. Given their cell wall- and membrane stress response phenotypes, it is tempting to speculate that Pi-sparing modifications of membranes and the cell wall might increase fitness during Pi scarcity. The cyanobacterium *Prochlorococcus*, a dominant species in waters of the North Pacific Subtropical Gyre, has a competitive advantage in this Pi-scarce ecosystem: its membrane is largely composed of sulfo- and glycolipids, in which fatty acids are linked to sulfate/sugar- or sugar-based, instead of phosphate-based, polar head groups [96]; other phytoplankton use similar adaptations [4]. *C*. *albicans* also economizes on non-essential uses of Pi by remodeling its membrane systems: *BTA1*, the gene encoding a homolog of diacylglyceryl-*N*,*N*,*N*-trimethylhomoserine synthase is strongly upregulated by the PHO regulon during Pi starvation [64] and like *Neurospora crassa* and *C*. *neoformans*, *C*. *albicans* can replace membrane phospholipids with betaine-headgroup lipids during Pi starvation [71,97].

A caveat is that the distinct stress phenotypes of the two passage 30 populations could be incidental or they could be integral to their Pi management strategies. Testing causal relationships between phenotypes will require detailed genotype comparisons and further genetic and cell biologic analysis of distinct mutations in the two lineages. For example, distinct SDS stress phenotypes and distinct strategies to Pi scarcity adaptation could be unrelated. Alternatively, the overall genomic changes that occurred in Q- L2 during Pi scarcity adaptation may have led to membrane changes that rendered it susceptible to detergent stress from SDS. In contrast, the adaptation trajectory of Q- L1 may have required less changes to membrane composition so that its SDS endurance was restored as it adapted to growth in scarce Pi. Genetic, genomic and lipidomic analyses will be required to distinguish these possibilities.

Except for rapamycin resistance of the Q- L2 population, evolved Q- populations did not surpass fitness of *pho84*-/- cells in any nutritional or stress conditions (Fig 8). This observation raises the question whether Pho84 has roles in stress management that are separable from its role in Pi uptake. For example, we previously found that *pho84*-/- cells contain excess levels of DCFDA-detectable reactive oxygen species (ROS) [26]. When these cells overexpress the TORC1-activating small GTPase Gtr1, their ROS levels normalize; in this context, activating TORC1 can apparently suppress the absence of a function in oxidative stress management provided by Pho84. Whether specific stress phenotypes of Q- strains and their evolved descendants, that do not recover beyond *pho84*-/- phenotypes, are related to Pho84 signaling functions, remains to be determined.

Using SnpEff [73] to predict potential large-impact mutations in both evolving lineages that were retained in at least one lineage by the end of the experiment, we identified *PTP2* and *FET99*. The predicted tyrosine phosphatase Ptp2 sustains hyphal growth in response to decreased TORC1 signaling by dephosphorylating Hog1 and thereby decreasing its basal activity [74]. In addition to its role in hyphal growth, Ptp2 appears to participate in cell wall stress responses: binding of the transcriptional regulator Sko1 in the intergenic region of *PTP2* is enriched during caspofungin exposure [98], suggesting *PTP2* transcription is regulated by Sko1 in response to cell wall stress. The frameshift in *PTP2* we noted in Q- L1 might perturb Hog1 dephosphorylation. A resulting basal upregulation of Hog1 could recalibrate stress responses of Q- L1. To substantiate this idea, genetic experiments are required, e.g. introducing a wild type copy of *PTP2* into representative clones from P30 of both lineages, and CRISPR-mutagenizing the same 2-nucleotide deletion into the ancestral Q- isolates of both lineages. A role of Fet99 in adaptation to Pi limitation might be linked to plasma membrane properties determined by ergosterol abundance. 14-alpha demethylase or Erg11, a crucial ergosterol biosynthetic enzyme, requires iron sufficiency because of its heme dependence, and a heme-binding protein, Dap1, links iron homeostasis with Erg11 activity in *C*. *albicans* [99] and *C*. *glabrata* [100]. Hence, iron uptake, ergosterol biosynthesis and plasma membrane properties could be connected through Fet99. Here again, mechanistic speculation must be tested by genetic and biochemical experiments as for *PTP2*. Of note, mutants in genes linked to TORC1 emerged both in our analysis of the most persistent trisomy among the evolving populations, and of large-impact mutations affecting both lineages that are retained by the end of the experiment.

Validation experiments are also required to examine a role of specific genes within the ~300 kb trisomic region of Chr2 that persisted in Q- L2 through the end of the in vitro evolution experiment (Fig 9 and S1 Table). We noticed that in addition to a gene potentially involved in uptake of an organic phosphate compound, *GIT1*, this region comprises 2 genes related to TORC1 function, encoding the Tor1 kinase itself and the Hog1 MAP kinase-activated protein kinase Rck2 (S1 Table); *C*. *albicans rck2* null mutants are hypersensitive to rapamycin [72]. To test a role of an increased copy number of these genes and their resulting predicted overexpression in adaptation to limited Pi, they will need to be expressed from a conditional element like *tetO* in the two progenitor Q- strains, singly and in combination.

Going forward, our goal is to examine the functional contribution of variants detected by WGS. A possible approach is to compare genome-wide expression in WT, Q- L1 and -L2 cells, selected passage populations and P30 of both lineages using RNA-Seq. Expression profiles of strains grown under conditions of scarce Pi that maximize their phenotypic differences, versus high Pi in which their phenotypic differences are minimized (Fig F in S1 Text) can aid in identifying the genomic variants that contribute to fitness during Pi scarcity. Since late-passage populations did not lose fitness in Pi-rich media as they regained fitness in Pi-limited medium (Fig F in S1 Text), this analysis can begin with genes differentially transcribed between the 2 evolved populations during growth in low but not in high Pi. These results can be compared with genes differentially transcribed between evolving populations and their progenitor mutant strains in low Pi. Gene Set Enrichment Analysis [101–103] of transcriptomic datasets can highlight pathways with divergent regulation between the lineages.

Differentially transcribed genes can be mapped back to genomic variants in regulatory regions, in open reading frames (e.g. corresponding to nonsense mutations), and in variants of their transcriptional regulators. Correlation of diverging stress phenotypes between the 2 lineages with transcriptomic findings under stress conditions, can further correlate genomic variants with phenotypic behaviors. Transcriptomic GSEA datasets can be combined with our WGS analysis of variant impacts. The product is a GSEA-informed dataset of mutations predicted to produce fitness effects under specific conditions like Pi limitation or cell wall stress. These descriptive analyses must then be confirmed genetically, by restoring wild type alleles of candidate mutant genes in clones of the evolved populations and by introducing selected combinations of variants into the progenitor Q- strains. Such a three-part analysis, of gene expression profiles and GSEA, variant impact correlation, and genetic confirmation, can be iterated across stress conditions to determine which stress phenotypes are mechanistically linked to fitness recovery in Pi limitation, and which are incidental products of chance [67].

The experiments reported here have several limitations. As noted, there was a residual Pi transport activity in addition to the predicted Pi transporters present in triple mutant cells due to presence of GPC transporters Git3/4; this residual activity was minor, though, and only detectable at high ambient Pi and acidic pH. Our experiments permit only semi-quantitative conclusions about the efficiency and optimum of each transporter, since we did not assay binding affinity and maximal transport velocity with ^32^P uptake measurements. Pi uptake experiments in early timepoints are likely not confounded by different growth rates of each triple mutant expressing a single Pi transporter “alone” because all experiments were performed at an OD_600_ of 2. However, inefficient Pi uptake may be artifactually amplified by slower growth of mutants at time points longer than 4–6 hours. Another potential confounder could be differential expression levels of the 4 transporters, which are likely to vary between pH and Pi concentration conditions; e.g. the alkali-responsive transcriptional regulator Rim101 induces transcription of *PHO89* [104], and protein levels of these 4 *C*. *albicans* transporters remain to be defined. Our in vitro evolution experiment was limited in that 2 transformants from a final strain-construction transformation step were evolved without technical replicates; nevertheless, experimental testing of candidate variants will be required in any case to ascribe fitness roles in Pi scarcity to mutations identified in both or in one of the phenotypically distinct lineages.

In summary, *C*. *albicans’* Pi acquisition system is suboptimal for the neutral to alkaline host environments that the pathogen typically occupies during invasive disease. Differences in functional optima among transporters may provide backup mechanisms for Pi transport as *C*. *albicans* moves through different host niches. Redistribution of intracellular Pi among organelles and processes may sustain survival during “alkaline pH-simulated nutrient deprivation” [79], in ways that remain to be elucidated. At the same time, this redistribution might also render the fungus more sensitive to host-relevant stressors like membrane- and cell wall stress. Pi acquisition and regulation in humans differs fundamentally from that in fungi; given the crucial role of phosphorus in structural, metabolic and regulatory processes and in antifungal drug endurance, definition of these systems could reveal potential fungus-specific drug targets.

## Methods

### Culture conditions

Cells were grown in rich complex medium, YPD, synthetic complete (SC) or yeast nitrogen base (YNB) with 2% glucose as described in [26,27]. To provide defined Pi concentrations, Yeast Nitrogen Base without amino acids, without ammonium sulphate and without phosphate, supplemented with KCl, was used (CYN6802, Formedium, Swaffham, Norfolk, England) and supplemented with the indicated concentrations of KH_2_PO_4_. Incubation temperatures were 30°C for liquid and solid media except for hyphal growth assays which were incubated at 37°C. For solid and liquid media with defined pH, 100 mM 2-(*N*-morpholino) ethanesulfonic acid (MES) was used to buffer the medium to the desired pH except for the filamentation assay (Fig 2) where 165 mM MOPS was used to adjust RPMI medium to pH 7.

### Strain construction

*C*. *albicans* mutants were generated as in [105]; details of strain genotypes and their construction are provided in S2 Table (Strains used in this study), S3 Table (Plasmids used in this study) and S4 Table (Oligonucleotides used in this study). Integration of mutation-inducing constructs was confirmed by PCR at the upstream and downstream junctures of the chromosome with the predicted integrated mutation-inducing construct, and sequencing of PCR products. Once integration of our FLP-*NAT1* containing mutation-inducing constructs was confirmed, expression of flippase was induced in YNB-BSA medium (23.4 g yeast carbon base, 4 g bovine serum albumin per liter, pH 4) with 2% maltose as described in [106] for 1–2 days at 30°C. Cell dilutions producing single colonies were then plated on YPD and, upon colony growth, replicaplated onto YPD with 200 μg/ml nourseothricin. Colonies unable to grow on nourseothricin were picked and tested by PCR. At each mutant construction step, clones whose correct integration of the mutation-inducing construct was confirmed were subjected to phenotype analysis and, if their phenotypes coincided with those of the majority of other clones of the same genotype, were saved for further use, while clones with outlier phenotypes (possibly containing significant transformation-induced mutations) were discarded. At least 2 and typically 3 independent heterozygous lineages were constructed for each set of deletion mutants. Only during construction of the Q- strain, 2 isolates, JKC2830 and JKC 2860, were generated from a single *pho84-/- pho89-/- pho87-/- fgr2/FGR2* heterozygous parent. Among isogenic mutants with similar phenotypes, we performed initial phenotypic characterizations with 2 or more isolates to confirm their similar behaviors. The null mutants for each predicted transporter were used to examine the role of that transporter in filamentous growth, stress responses, susceptibility to antifungal agents and TORC1 signaling.

### Construction of strains with multiple mutations

Plasmids and oligonucleotides are detailed in S3 and S4 Tables.

Construction of the Pho84-Alone strain (*pho87-/- pho89-/- fgr2-/-* triple mutant): Wild type strain JKC915 [105] was transformed with pJK1372 and pJK1479 to sequentially delete two alleles of *PHO87*, resulting in JKC2581 (*pho87-/-*). JKC2581 was then transformed with pJK1384 and pJK1481 to sequentially delete two alleles of *PHO89*, resulting in JKC2679 (*pho87-/- pho89-/-*). JKC2679 was then transformed with pJK1485 and pJK1488 to sequentially delete two alleles of *FGR2*, resulting in JKC2788 (*pho87-/- pho89-/- fgr2-/-*).

Construction of the Pho87-Alone strain (*pho84-/- pho89-/- fgr2-/-* triple mutant): JKC1450 (*pho84-/-*) [8] was transformed with pJK1384 and pJK1481 to sequentially delete two alleles of *PHO89*, resulting in JKC2592 (*pho84-/- pho89-/-*). JKC2592 was then transformed with pJK1485 and pJK1488 to sequentially delete two alleles of *FGR2*, resulting in JKC2777 (*pho84-/- pho89-/- fgr2-/-*).

Construction of the Pho89-Alone strain (*pho84-/- pho87-/- fgr2-/-* triple mutant): JKC1450 (*pho84-/-*) [8] was transformed with pJK1372 and pJK1479 to sequentially delete two alleles of *PHO87*, resulting in JKC2599 (*pho84-/- pho87-/-*). JKC2599 was then transformed with pJK1485 and pJK1488 to sequentially delete two alleles of *FGR2*, resulting in JKC2783 (*pho84-/- pho87-/- fgr2-/-*).

Construction of the Fgr2-Alone strain (*pho84-/- pho87-/- pho89-/-* triple mutant): JKC1450 (*pho84-/-*) [8] was transformed with pJK1372 and pJK1479 to sequentially delete two alleles of *PHO87*, resulting in JKC2599 (*pho84-/- pho87-/-*). JKC2599 was then transformed with pJK1384 and pJK1481 to sequentially delete two alleles of *PHO89*, resulting in JKC2758 (*pho84-/- pho87-/- pho89-/-*).

Construction of quadruple mutants: Using quadruple mutant strain JKC2830 as an example, JKC1450 (*pho84-/-*) [8] was transformed with pJK1372 and pJK1479 to sequentially delete two alleles of *PHO87*, resulting in JKC2599 (*pho84-/- pho87-/-*). JKC2599 was then transformed with pJK1384 and pJK1481 to sequentially delete two alleles of *PHO89*, resulting in JKC2758 (*pho84-/- pho87-/- pho89-/-*). JKC2758 was then transformed with pJK1485 and pJK1488 to sequentially delete two alleles of *FGR2*, resulting in JKC2830 (*pho84-/- pho87-/- pho89-/- fgr2-/-*).

Construction of septuple mutants, not constructed from Q- strains but sequentially from the *pho84* null mutant: Using septuple mutant strain JKC2969 as an example, JKC1450 (*pho84-/-*) [8] was transformed with pJK1384 and pJK1481 to sequentially delete two alleles of *PHO89*, resulting in JKC2592 (*pho84-/- pho89-/-*). JKC2592 was then transformed with pJK1485 and pJK1488 to sequentially delete two alleles of *FGR2*, resulting in JKC2773 (*pho84-/- pho89-/- fgr2-/-*). JKC2773 was first transformed with pJK1375 to place one allele of *PHO87* under *tetO* control, then transformed with pJK1372 to delete the WT allele of *PHO87*, resulting in JKC2804 (*pho84-/- pho89-/- fgr2-/- pho87/tetO-PHO87*). JKC2804 was transformed with pJK1543 and pJK1545 to sequentially delete two alleles of *GIT2-4*, resulting in JKC2969 (*pho84-/- pho89-/- fgr2-/- tetO-PHO87/pho87 git2-4-/-*).

### Growth curves

Cells from glycerol stocks at -80°C were recovered on YPD agar medium for 2 days. Cells were scraped from the plate, washed twice with 0.9% NaCl and diluted to a final OD_600_ 0.01 in 150 μl medium in flat bottom 96-well plates. OD_600_ readings were obtained every 15 min in a Biotech Synergy 2 Multi-Mode Microplate Reader (Winooski, VT, USA). Standard deviations of three technical replicates, representing separate wells, were calculated, and graphed in Graphpad Prism Version 9.5.1 (528), and displayed as error bars. At least 3 biological replicates were obtained on different days unless stated otherwise. For some assays the area under the curve (AUC) was calculated and graphed using the same software, and the average from ≥3 biological replicates per condition was graphed.

### Phosphate uptake

Phosphate uptake measurement experiments were performed as in [25] with some modifications. Cells of indicated genotypes were recovered from -80°C on a YPD plate for ~40 hours. SC medium without Pi, buffered at one-unit increments from pH 2 to 8 with 100 mM MES was inoculated with cells at an OD_600_ of 2. Cells were given 30 minutes to equilibrate, 1 mM Pi was then added, and the Pi remaining in the medium was measured every 1–5 h for a period of 6 to 30 hours, depending on each strain or condition. Samples were harvested at 20,000 rpm for 5 min in the cold room and two technical replicates per sample (300 μl) were collected from each time point. Pi standards containing 18.75 μM, 37.5 μM, 75 μM, 150 μM, 300 μM phosphate were prepared by diluting a 17.2 M phosphoric acid stock (density 1.69 g/ml, Mw 98 g/mol) in ddH_2_O.

The total concentration of Pi in the supernatant was calculated according to Ames [107]. In brief, 700 μl Pi assay mix (1 part of 10% w/v ascorbic acid mixed with 6 parts of 0.42% w/v ammonium molybdate in 1N sulfuric acid, prepared freshly and kept on ice during the day) was added to each 300 μl culture supernatant or Pi standard, and incubated at 37°C for 1 h. Absorption at 700 nm was measured at the end of the incubation and a Pi concentration calibration curve was generated using Pi standards. The Pi concentration of each sample was then calculated from the calibration curve and plotted.

When the assay required pre-feeding the cells GPC before the addition of phosphate, to test the role of Git transporters in the uptake of Pi in the quadruple null mutant, GPC was added to a concentration of 10 mM at the beginning of the 30 minute incubation without Pi. Then Pi was added to a concentration of 1 mM.

### Growth of cell dilution spots on solid medium

Cells recovered from glycerol stock at -80°C were grown on YPD plates for 2 days. They were washed in 0.9% NaCl and diluted in 5- or 3-fold steps from a starting OD_600_ of 0.5 in a microtiter plate, then pin transferred to agar medium and incubated for 48 h at 30°C.

To quantitate the spotting assays as mutant strains’ relative growth to WT, individual spot images were processed in ImageJ software. Each image’s color (black and white) was first inverted to remove background, then inverted back (to restore black background) to measure spot areas’ integrated signal intensity using ImageJ’s built-in measuring function. The spots’ integrated signal intensity ratio of each strain vs. WT control on the same plate was then calculated and plotted in Graphpad Prism Version 9.5.1.

Statistical analysis was done using Graphpad Prism Version 9.5.1’s unpaired two-tailed t-test. For statistical significance: ns, *p* > 0.05; *, 0.01 < *p* ≤ 0.05; **, 0.001 < *p* ≤ 0.01; ***, 0.0001 < *p* ≤ 0.001; ****, 0.00001 < *p* ≤ 0.0001.

### Hyphal morphogenesis assay

Cells recovered from glycerol stock at -80°C were grown on YPD plates for 1–2 days, washed and resuspended in 0.9% NaCl to an OD_600_ 0.1. Variation between spots and spot density effects were minimized by spotting 3 μl cell suspensions at 6 equidistant points, using a template around the perimeter of an agar medium plate. Each agar plate contained a WT spot that served as a control to which the other strains on the plate must be compared. This method minimizes variation between colony filamentation within each genotype that occurs when colonies are streaked or plated at varying density and at uncontrolled distances from each other. By including a WT on each plate, we also control for the effects of different hydration states of the agar and slight variations in medium composition which cannot be excluded by other means. RPMI and Spider media were used; the latter is not buffered and has a slightly higher than neutral pH. RPMI medium pH 7 was buffered with 165 mM MOPS; and RPMI pH 5 was buffered with 100 mM MES. Plates were incubated at 37°C for the indicated durations. For photomicrography, spot edges were aligned with image frame corners to allow comparisons of hyphal fringes’ length.

### Western blot

Cell lysis and Western blotting were performed as described in [65]. Antibodies used are shown in S5 Table. For densitometry, ImageJ (imagej.net/welcome) software (opensource) was used to quantitate signals obtained from Azure biosystems c600.

### Population evolution

Two Q- mutants (*pho84-/- pho89-/- pho87-/- fgr2-/-*), distinct transformants (Q- L1 and Q- L2) for deletion of the second *FGR2* allele, derived from the same heterozygous strain (JKC2812 *pho84-/- pho89-/- pho87-/- fgr2-/FGR2*), were inoculated into 10 ml SC 0.4 mM Pi at an OD_600_ of 0.05. Cultures were incubated at 30°C at 200 rpm. From each culture, 10 μl were transferred into 10 ml of the same fresh medium every 48 hours, for a total of 30 passages. The culture from each passage was saved as a glycerol stock. Populations from each passage were used in growth curve experiments and were always used after direct revival from frozen stock without further passaging.

### Genomic DNA isolation and whole-genome sequencing

For DNA extraction from cells grown on YPD plates from cells saved at the end of each passage, the Zymo Quick DNA Fungal/Bacterial Miniprep kit was used according to the manufacturer’s instructions. Library preparation and sequencing was carried out by the Applied Microbiology Services Lab (AMSL) at The Ohio State University, using the Illumina Nextseq 2000 platform to generate 150 basepair paired-end reads. All samples were sequenced to a minimum depth of 175x. The reads were trimmed using trimmomatic 0.39 (with default parameters except slidingwindow:4:20, maxinfo:125:1, headcrop:20, and minlen:35) to remove adaptors and poor quality sequences [108]. Using bowtie2 v2.2.6 [109], the trimmed reads were aligned against the SC5314 reference genome (version A21-s02-m09-r10) obtained from the Candida Genome Database (www.candidagenome.org). The aligned SAM files were then converted to the BAM format using samtools v1.7 [110].

### Copy number determination and variant calling

To detect karyotypic changes, pileup data for each whole-genome sequenced strain was obtained using bbMAP v39.01 [111]. Average read pileup depth highlighted regions of various copy number. Copy number variation, including aneuploidies, were further confirmed via visualization in YMAP [85].

The Genome Analysis Toolkit (GATK4) [112];[113] was used to call variants (SNPs and INDELs) from the SC5314 reference-aligned BAM files. The reads were preprocessed using Picard Tools (http://broadinstitute.github.io/picard/; AddOrReplaceReadGroups, MarkDuplicates, CreateSequenceDictionary, and ReorderSam). Variant calling and filtration were done using the GATK4 germline short variant discovery workflow with HaplotypeCaller following the GATK4 best practices (parameters for filtering out reads: QD < 2.0, FS > 60.0, MQ < 40.0, MQRankSum < –12.5, ReadPosRankSum < −8, QUAL < 50, DP < 20). The resultant VCF files were then used to compare variants between evolved lineages and time points, using bcftools v1.10.2 [114] and SnpEff v5.1 [73].

## Supporting information

S1 Text Supplemental figures. **Fig A in S1 Text. Structural comparisons of Pho84 and its homologs.** AlphaFold structure prediction of *S*. *cerevisiae* Pho84 and *C*. *albicans* Fgr2 was obtained from the AlphaFold Protein Structure Database (https://alphafold.ebi.ac.uk/) and visualized in ChimeraX-1.4. The coloring of each model is based on a per-residue confidence score (pLDDT): dark blue–very high (pLDDT > 90), light blue–confident (90 > pLDDT > 70), yellow–low (70 > pLDDT > 50), orange–very low (pLDDT < 50). Pi transporters were aligned in MacVector; identical amino acid residues are tinted in dark gray and chemically similar ones in light gray. **Fig B in S1 Text. Among Pi transporters, only Pho84 was required for oxidative stress endurance.** Cell suspensions of the indicated genotypes WT (JKC915); *pho84-/-* (JKC1450); *pho87-/-* (JKC2581); *pho89-/-* (JKC2585); *fgr2-/-* (JKC2667) and *git2-4-/-* (JKC2963) were spotted in 3-fold dilution steps onto SC medium with DMSO (Veh) or plumbagin 15 μM. Plates were incubated for 2 days at 30°C. Representative of 3 biological replicates. All spots were on the same plate. **Fig C in S1 Text. Individual growth curves summarized in Fig 4 and Fig D in S1 Text.** Strains were grown as described in Fig 4. Shown are 3 biological replicates performed on different days. Error bars SD of 2 or 3 technical replicates. Strains are WT (JKC915); Pho84-A: *pho87-/- pho89-/- fgr2-/- PHO84+/+* (JKC2788); Pho87-A: *pho84-/- pho89-/- fgr2-/- PHO87+/+* (JKC2777); Pho89-A: *pho84-/- pho87-/- fgr2-/- PHO89+/+* (JKC2783); Fgr2-A: *pho84-/- pho87-/- pho89-/- FGR2+/+* (JKC2758). **Fig D in S1 Text. Growth of strains containing a single Pi transporter represented as Area Under the growth Curve as in Fig 4, grouped by pH.** Strains were grown as described in Fig 4. Shown is the area under the growth curve grouped by pH. Error bars SD of 3 biological replicates. Strains are WT (JKC915); Pho84-A: *pho87-/- pho89-/- fgr2-/- PHO84+/+* (JKC2788); Pho87-A: *pho84-/- pho89-/- fgr2-/- PHO87+/+* (JKC2777); Pho89-A: *pho84-/- pho87-/- fgr2-/- PHO89+/+* (JKC2783); Fgr2-A: *pho84-/- pho87-/- pho89-/- FGR2+/+* (JKC2758). **A.** SC containing 0.1 mM KH_2_PO4. **B.** SC containing 7.3 mM KH_2_PO4. **A, B**: Pho84-A vs WT: no significant difference observed at pH 2–8. For statistical significance: ns, *p* > 0.05; *, 0.01 < *p* ≤ 0.05; **, 0.001 < *p* ≤ 0.01; ***, 0.0001 < *p* ≤ 0.001; ****, 0.00001 < *p* ≤ 0.0001 by Student’s 2-tailed t-test. **Fig E in S1 Text. Pi transporter triple mutants had no growth defects in rich complex medium.** Cells of indicated triple mutant genotypes were grown in YPD (left) and SC (right) and OD_600_ was monitored. Upper panels: strains expressing only one of 2 high-affinity transporters. Lower panels: Strains expressing only one of 2 low-affinity transporters. Pho84-A: *pho87-/- pho89-/- fgr2-/- PHO84+/+* (JKC2788); Pho89-A: *pho84-/- pho87-/- fgr2-/- PHO89+/+* (JKC2783); Pho87-A: *pho84-/- pho89-/- fgr2-/- PHO87+/+* (JKC2777); Fgr2-A: *pho84-/- pho87-/- pho89-/- FGR2+/+* (JKC2758). Representative of 3 biological replicates; error bars SD of 3 technical replicates. **Fig F in S1 Text. *tetO-PHO87* cells, Q- cells and their Pi scarcity-evolved descendant populations had no substantial growth defects in rich complex medium.** Strains were grown as in Fig E in S1 Text. **A.** Cells in which a single allele of one Pi transporter, *PHO87*, is expressed from repressible *tetO*, were grown in YPD and SC without doxycycline and compared with WT and Q- cells. WT (JKC915), Q- L1 (*pho84-/- pho89-/- pho87-/- fgr2-/-*, JKC2830), *tetO-PHO87* (*tetO-PHO87/pho87 pho84-/- pho89-/- fgr2-/- git2-4-/-*, JKC2969). **B.** Growth in YPD of WT (JKC915); Q- L1 (JKC2830) and Q- L2 (*pho84-/- pho89-/- pho87-/- fgr2-/-*, JKC2860). P30: population from the 30^th^ Pi scarcity passage.
(PDF)

S1 Table Annotation of open reading frames within the Chr2 triploid segment of Q- L2 P30. (XLSX)

S2 Table Strains used in this study and construction of strains with multiple mutations. (PDF)

S3 Table Plasmids used in this study. (PDF)

S4 Table Oligonucleotides used in this study. (PDF)

S5 Table Antibodies used in this study. (PDF)

S6 Table Raw data Fig 1 growth quantification. (XLSX)

S7 Table Raw data Fig 3 Western Blot densitometry. (XLSX)

S8 Table Raw data Fig 4 pH2-8 AUC calculation. (XLSX)

S9 Table Raw data Fig 5 Pi uptake raw data and histogram. (XLSX)

S10 Table Raw data Fig 6 Pi uptake and growth curve. (XLSX)

S11 Table Raw data Fig 7 Q- growth curves and AUC. (XLSX)

S12 Table Raw data Fig 8 growth curves AUC. (XLSX)

S13 Table Raw data Fig C in S1 Text individual growth curves. (XLSX)

S14 Table Raw data Fig D in S1 Text growth curves. (XLSX)

S15 Table Raw data Fig E in S1 Text growth curves. (XLSX)
