# Supplementary material for: Candida albicans’ inorganic phosphate transport and evolutionary adaptation to phosphate scarcity
Source: PLoS Genet. 2024 Aug 13;20(8):e1011156. doi: 10.1371/journal.pgen.1011156 (PMC11343460; doi:10.1371/journal.pgen.1011156)
Supplement: S1 Text — Fig A in S1 Text. Structural comparisons of Pho84 and its homologs. AlphaFold structure prediction of S. cerevisiae Pho84 and C. albicans Fgr2 was obtained from the AlphaFold Protein Structure Database (https://alphafold.ebi.ac.uk/) and visualized in ChimeraX-1.4. The coloring of each model is based on a per-residue confidence score (pLDDT): dark blue–very high (pLDDT > 90), light blue–confident (90 > pLDDT > 70), yellow–low (70 > pLDDT > 50), orange–very low (pLDDT < 50). Pi transporters were aligned in MacVector; identical amino acid residues are tinted in dark gray and chemically similar ones in light gray. Fig B in S1 Text. Among Pi transporters, only Pho84 was required for oxidative stress endurance. Cell suspensions of the indicated genotypes WT (JKC915); pho84-/- (JKC1450); pho87-/- (JKC2581); pho89-/- (JKC2585); fgr2-/- (JKC2667) and git2-4-/- (JKC2963) were spotted in 3-fold dilution steps onto SC medium with DMSO (Veh) or plumbagin 15 μM. Plates were incubated for 2 days at 30°C. Representative of 3 biological replicates. All spots were on the same plate. Fig C in S1 Text. Individual growth curves summarized in Fig 4 and Fig D in S1 Text. Strains were grown as described in Fig 4. Shown are 3 biological replicates performed on different days. Error bars SD of 2 or 3 technical replicates. Strains are WT (JKC915); Pho84-A: pho87-/- pho89-/- fgr2-/- PHO84+/+ (JKC2788); Pho87-A: pho84-/- pho89-/- fgr2-/- PHO87+/+ (JKC2777); Pho89-A: pho84-/- pho87-/- fgr2-/- PHO89+/+ (JKC2783); Fgr2-A: pho84-/- pho87-/- pho89-/- FGR2+/+ (JKC2758). Fig D in S1 Text. Growth of strains containing a single Pi transporter represented as Area Under the growth Curve as in Fig 4, grouped by pH. Strains were grown as described in Fig 4. Shown is the area under the growth curve grouped by pH. Error bars SD of 3 biological replicates. Strains are WT (JKC915); Pho84-A: pho87-/- pho89-/- fgr2-/- PHO84+/+ (JKC2788); Pho87-A: pho84-/- pho89-/- fgr2-/- PHO87+/+ (JKC2777); Pho89-A: pho84-/- pho [file pgen.1011156.s001.pdf]

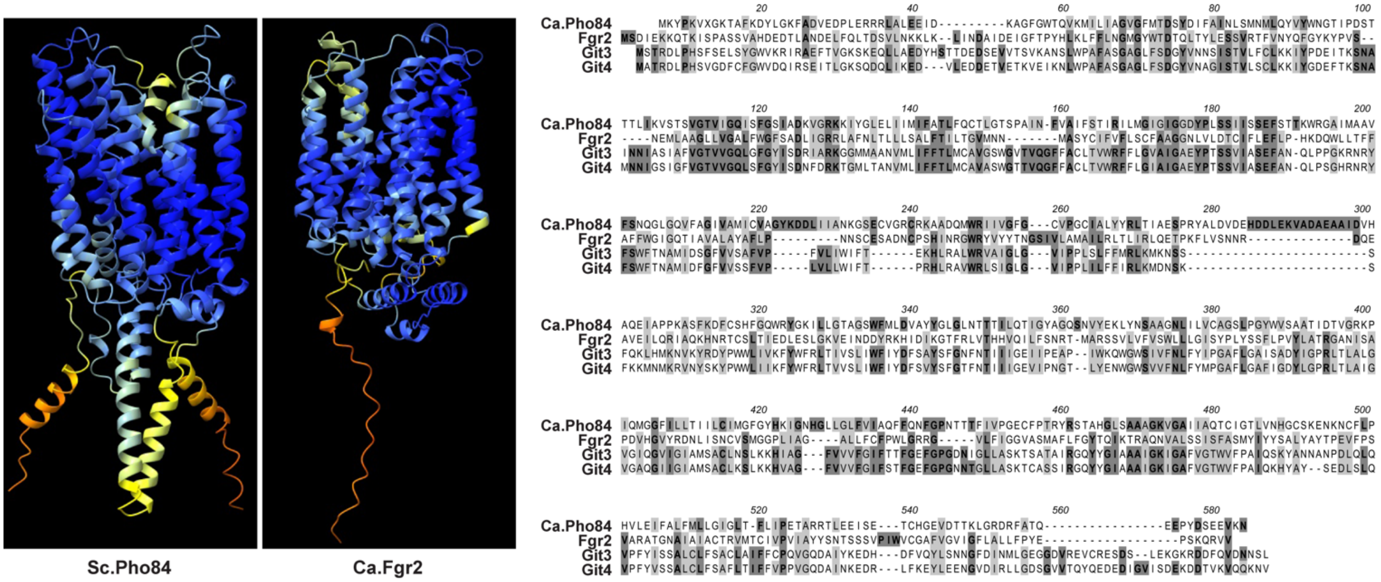

S1 Text Fig A. Structural comparisons of Pho84 and its homologs. AlphaFold structure prediction of *S. cerevisiae* Pho84 and *C. albicans* Fgr2 was obtained from the AlphaFold Protein Structure Database (https://alphafold.ebi.ac.uk/) and visualized in ChimeraX-1.4. The coloring of each model is based on a per-residue confidence score (pLDDT): dark blue – very high (pLDDT > 90), light blue – confident (90 > pLDDT > 70), yellow – low (70 > pLDDT > 50), orange – very low (pLDDT < 50). Inorganic phosphate transporters were aligned in MacVector; identical amino acid residues tinted with dark gray and chemically similar ones in light gray.

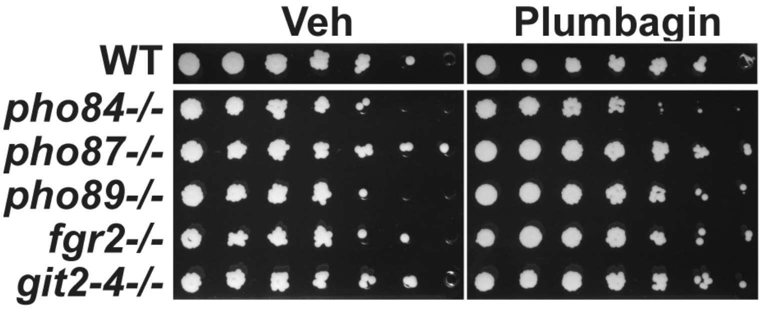

S1 Text Fig B. Among Pi transporters, only Pho84 was required for oxidative stress endurance. Cell suspensions of the indicated genotypes WT JKC915; *pho84-/-* (JKC1450); *pho87-/-* (JKC2581); *pho89-/-* (JKC2585); *fgr2-/-* (JKC2667) and *git2-4-/-* (JKC2963) were spotted in 3-fold dilution steps onto SC medium with DMSO (Veh) or plumbagin 15 µM. Plates were incubated for 2 days at 30° C. Representative of 3 biological replicates. All spots were on the same plate.

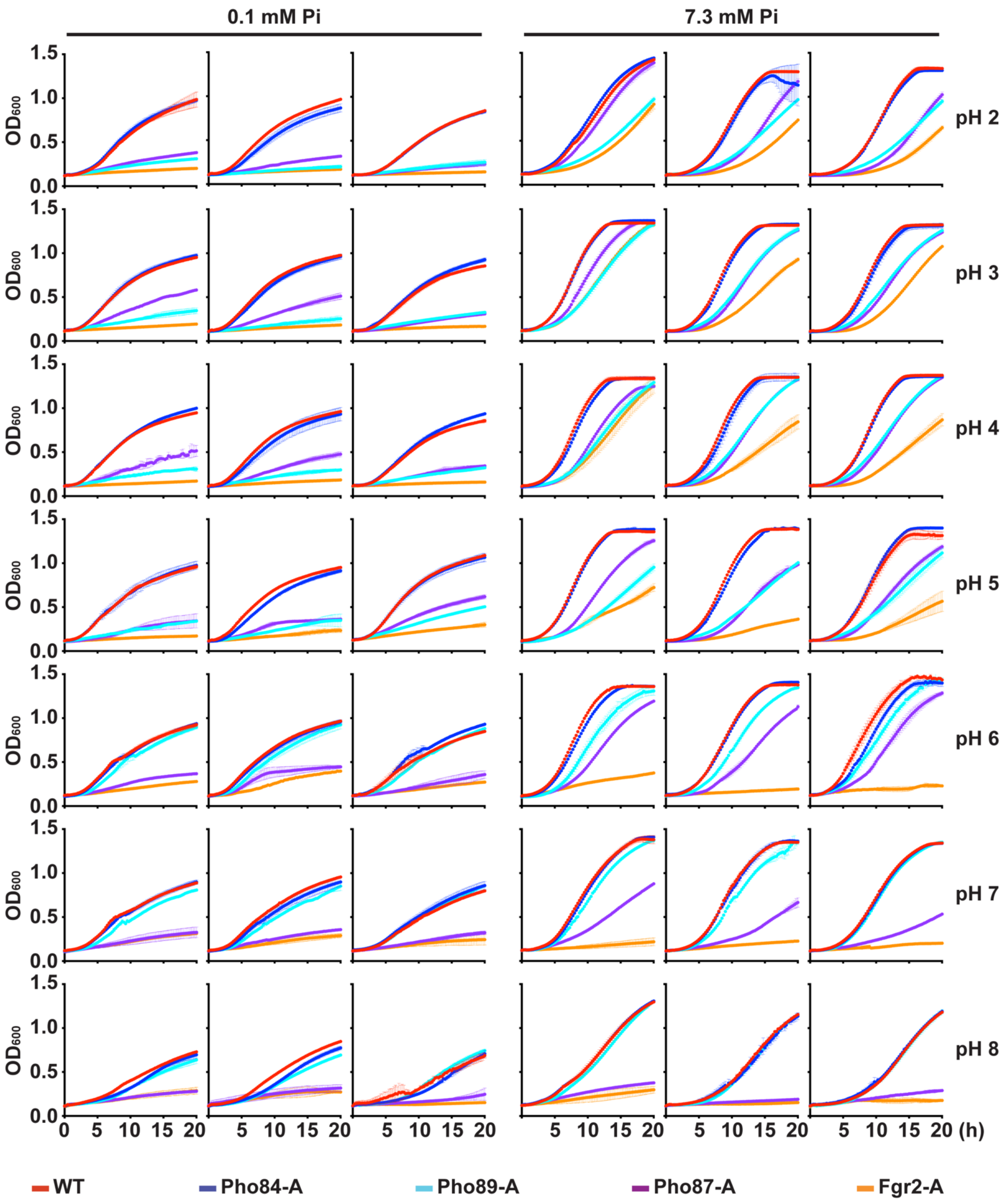

S1 Text Fig C. Individual growth curves summarized in Figure 4 and S1 Text Fig D. Strains were grown as described in Fig. 4. Shown are 3 biological replicates performed on different days. Error bars SD of 2 or 3 technical replicates. Strains are WT (JKC915); Pho84-A: *pho87-/- pho89-/- fgr2-/- PHO84+/+* (JKC2788); Pho87-A: *pho84-/- pho89-/- fgr2-/- PHO87+/+* (JKC2777); Pho89-A: *pho84-/- pho87-/- fgr2-/- PHO89+/+* (JKC2783)*;* Fgr2-A: *pho84-/- pho87-/- pho89-/- FGR2+/+* (JKC2758).

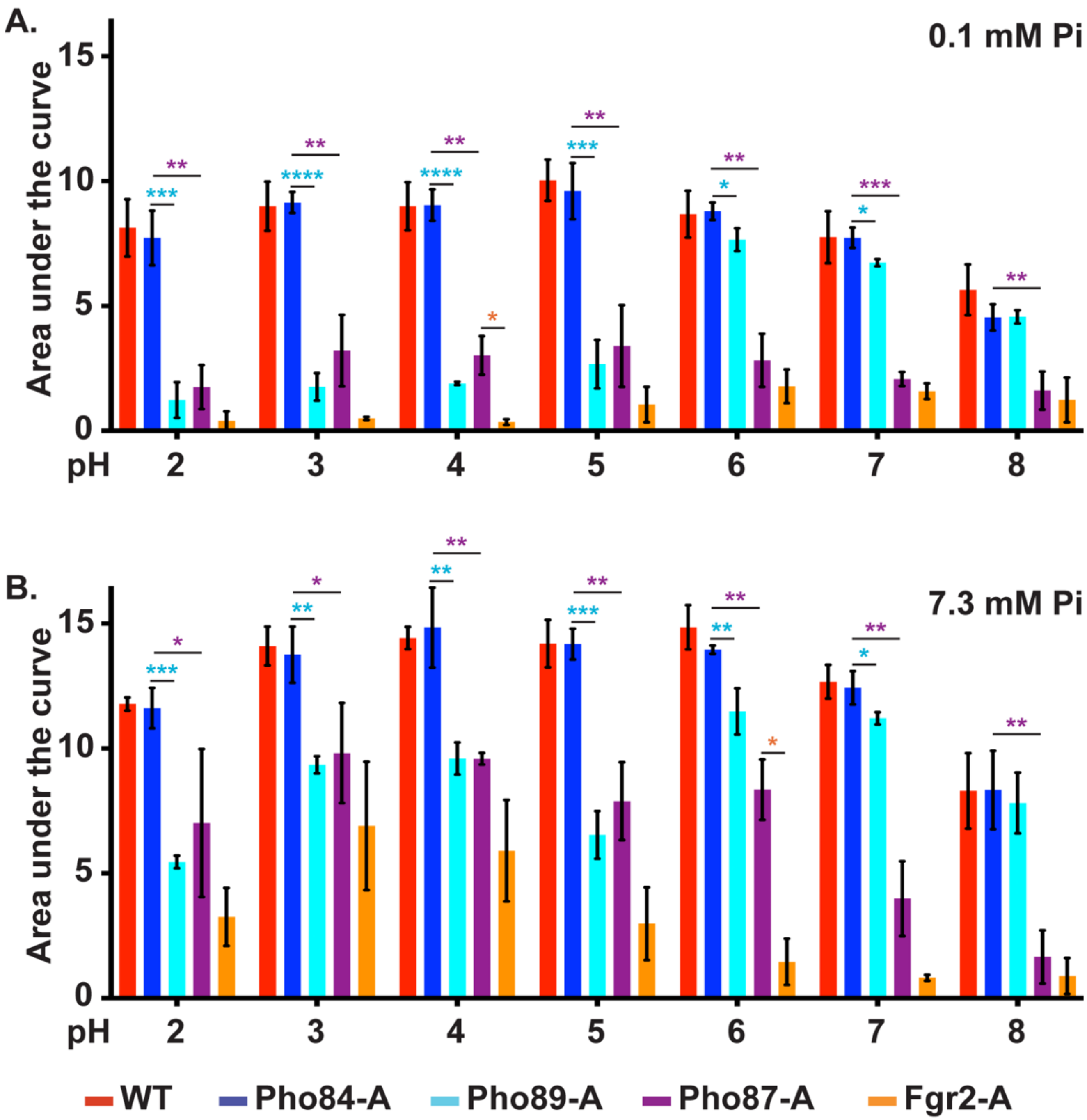

S1 Text Fig D. Growth of strains containing a single Pi transporter represented as Area Under the growth Curve as in Fig. 4, grouped by pH. Strains were grown as described in Fig. 4. Shown is the area under the growth curve grouped by pH. Error bars SD of 3 biological replicates. Strains are WT (JKC915); Pho84-A: *pho87-/- pho89-/- fgr2-/- PHO84+/+* (JKC2788); Pho87-A: *pho84-/- pho89-/- fgr2-/- PHO87+/+* (JKC2777); Pho89-A: *pho84-/- pho87-/- fgr2-/- PHO89+/+* (JKC2783)*;* Fgr2-A: *pho84-/- pho87-/- pho89-/- FGR2+/+* (JKC2758). **A.** SC containing 0.1 mM KH_2PO4. **B.** SC containing 7.3 mM KH_2PO4. **A, B**: Pho84-A vs WT: no significant difference observed at pH 2-8. For statistical significance: *, $0.01 < p \leq 0.05$; **, $0.001 < p \leq 0.01$; ***, $0.0001 < p \leq 0.001$; ****, $0.00001 < p \leq 0.0001$ by Student's 2-tailed t-test.

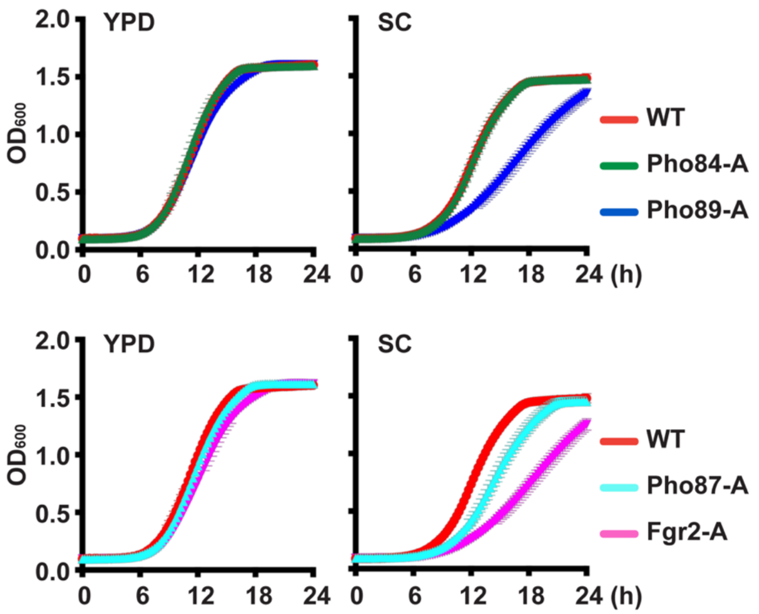

S1 Text Fig E. Pi transporter triple mutants had no growth defects in rich complex medium. Cells of indicated triple mutant genotypes were grown in YPD (left) and SC (right) and OD_{600} was monitored. Upper panels: strains expressing only one of 2 high-affinity transporters. Lower panels: Strains expressing only one of 2 low-affinity transporters. Pho84-A: *pho87-/- pho89-/- fgr2-/- PHO84+/+* (JKC2788); Pho89-A: *pho84-/- pho87-/- fgr2-/- PHO89+/+* (JKC2783); Pho87-A: *pho84-/- pho89-/- fgr2-/- PHO87+/+* (JKC2777); Fgr2-A: *pho84-/- pho87-/- pho89-/- FGR2+/+* (JKC2758). Representative of 3 biological replicates; error bars SD of 3 technical replicates.

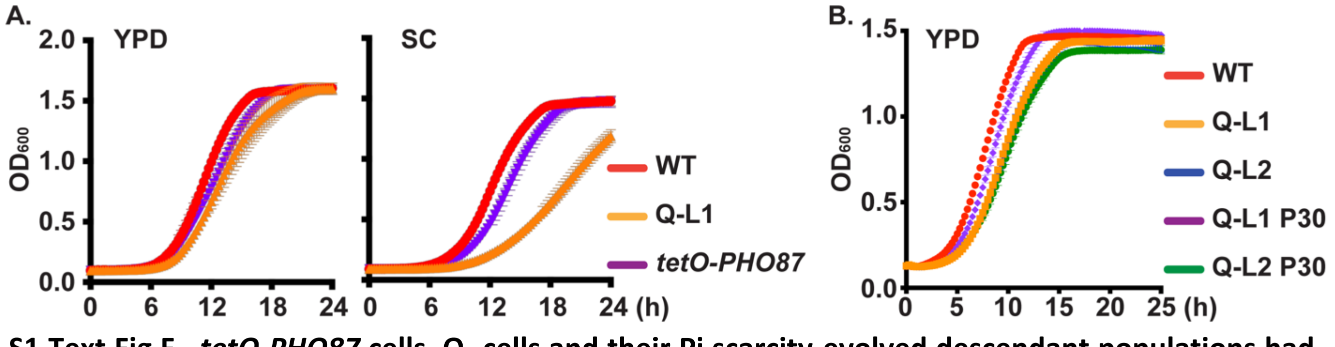

S1 Text Fig F. ***tetO-PHO87*** **cells, Q- cells and their Pi scarcity-evolved descendant populations had no substantial growth defects in rich complex medium.** Strains were grown as in S5 Fig. **A.** Cells in which a single allele of one Pi transporter, *PHO87*, is expressed from repressible *tetO*, were grown in YPD and SC without doxycycline and compared with WT and Q- cells. WT (JKC915), Q- L1 (*pho84-/- pho89-/- pho87-/- fgr2-/-*, JKC2830), *tetO-PHO87* (*tetO-PHO87/pho87 pho84-/- pho89-/- fgr2-/- git2-4-/-*, JKC2969). **B.** Growth in YPD of WT (JKC915); Q- L1 (JKC2830) and Q- L2 (*pho84-/- pho89-/- pho87-/- fgr2-/-*, JKC2860). P30: population from the 30th Pi scarcity passage.
