# Supplementary material for: Candida albicans’ inorganic phosphate transport and evolutionary adaptation to phosphate scarcity
Source: PLoS Genet. 2024 Aug 13;20(8):e1011156. doi: 10.1371/journal.pgen.1011156 (PMC11343460; doi:10.1371/journal.pgen.1011156)
Supplement: S2 Table — (PDF) [file pgen.1011156.s003.pdf]

S2 Table. Strains used in this study.

C. albicans strain name	Parent	Genotype	Strain construction	Reference
SC5314		Wild type		[1]
SN95		*arg4Δ/arg4Δ his1Δ/his1Δ IRO1/iro1Δ::λimm^{434} URA3/ura3Δ::λimm^{434}*		[2]
JKC915	SC5314	*HIS1/his1::tetR-FRT*		[3]
JKC1450	JKC1423	*pho84::HIS1/pho84::ARG4 his1/his1::tetR-FRT arg4/arg4 IRO1/iro1Δ::λimm^{434} URA3/ura3Δ::λimm^{434}*		[4]
JKC2579	JKC2566	*pho84::HIS1/pho84::ARG4 pho89::uPAM*-FRT/pho89::uPAM-FRT-FLP-NAT1 his1/his1::tetR-FRT arg4/arg4 IRO1/iro1Δ::λimm^{434} URA3/ura3Δ::λimm^{434}*	JKC2566 transformed with KpnI/BsiWI digested pJK1481 to delete the 2nd allele of *PHO89*.	This work
JKC2592	JKC2579	*pho84::HIS1/pho84::ARG4 pho89::uPAM-FRT/pho89::uPAM-FRT his1/his1::tetR-FRT arg4/arg4 IRO1/iro1Δ::λimm^{434} URA3/ura3Δ::λimm^{434}*	JKC2579 *NAT1* flipped out	This work
JKC2536	JKC915	*PHO87/pho87::uPAM-FRT-FLP-NAT1 HIS1/his1::tetR-FRT*	JKC915 transformed with KpnI/BsiWI digested pJK1372 to delete the 1st allele of *PHO87*.	This work
JKC2728	JKC2592	*pho84::HIS1/pho84::ARG4 pho89::uPAM-FRT/pho89::uPAM-FRT FGR2/fgr2::uPAM-FRT-FLP-NAT1 his1/his1::tetR-FRT arg4/arg4 IRO1/iro1Δ::λimm^{434} URA3/ura3Δ::λimm^{434}*	JKC2592 transformed with KpnI/BsiWI digested pJK1485 to delete the 1st allele of *FGR2*.	This work
JKC2548	JKC2536	*PHO87/pho87::uPAM-FRT HIS1/his1::tetR-FRT*	JKC2536 *NAT1* flipped out	This work
JKC2737	JKC2728	*pho84::HIS1/pho84::ARG4 pho89::uPAM-FRT/pho89::uPAM-FRT FGR2/fgr2::uPAM-FRT his1/his1::tetR-FRT arg4/arg4 IRO1/iro1Δ::λimm^{434} URA3/ura3Δ::λimm^{434}*	JKC2728 *NAT1* flipped out	This work
JKC2573	JKC2548	*pho87::uPAM-FRT/pho87::uPAM-FRT-FLP-NAT1 HIS1/his1::tetR-FRT*	JKC2548 transformed with KpnI/BsiWI digested pJK1479 to delete the 2nd allele of *PHO87*.	This work
JKC2596	JKC2554	*pho84::HIS1/pho84::ARG4 pho87::uPAM-FRT/pho87::uPAM-FRT-FLP-NAT1 his1/his1::tetR-FRT arg4/arg4 IRO1/iro1Δ::λimm^{434} URA3/ura3Δ::λimm^{434}*	JKC2554 transformed with KpnI/BsiWI digested pJK1479 to delete the 2nd allele of *PHO87*.	This work
JKC2764	JKC2737	*pho84::HIS1/pho84::ARG4 pho89::uPAM-FRT/pho89::uPAM-FRT fgr2::uPAM-FRT/fgr2::uPAM-FRT-FLP-NAT1 his1/his1::tetR-FRT arg4/arg4 IRO1/iro1Δ::λimm^{434} URA3/ura3Δ::λimm^{434}*	JKC2737 transformed with KpnI/BsiWI digested pJK1488 to delete the 2nd allele of *FGR2*.	This work
JKC2581	JKC2573	*pho87::uPAM-FRT/pho87::uPAM-FRT HIS1/his1::tetR-FRT*	JKC2573 *NAT1* flipped out	This work
JKC2599	JKC2596	*pho84::HIS1/pho84::ARG4 pho87::uPAM-FRT/pho87::uPAM-FRT his1/his1::tetR-FRT arg4/arg4 IRO1/iro1Δ::λimm^{434} URA3/ura3Δ::λimm^{434}*	JKC2596 *NAT1* flipped out	This work
JKC2773	JKC2764	*pho84::HIS1/pho84::ARG4 pho89::uPAM-FRT/pho89::uPAM-FRT fgr2::uPAM-FRT/fgr2::uPAM-FRT his1/his1::tetR-FRT arg4/arg4 IRO1/iro1Δ::λimm^{434} URA3/ura3Δ::λimm^{434}*	JKC2764 *NAT1* flipped out	This work
JKC2638	JKC2581	*pho87::uPAM-FRT/pho87::uPAM-FRT PHO89/pho89::uPAM-FRT-FLP-NAT1 HIS1/his1::tetR-FRT*	JKC2581 transformed with KpnI/BsiWI digested pJK1384 to delete the 1st allele of *PHO89*.	This work
JKC2545	JKC1450	*pho84::HIS1/pho84::ARG4 PHO89/pho89::uPAM-FRT-FLP-NAT1 his1/his1::tetR-FRT arg4/arg4 IRO1/iro1Δ::λimm^{434} URA3/ura3Δ::λimm^{434}*	JKC1450 transformed with KpnI/BsiWI digested pJK1384 to delete the 1st allele of *PHO89*.	This work
JKC2539	JKC1450	*pho84::HIS1/pho84::ARG4 PHO87/pho87::uPAM-FRT-FLP-NAT1 his1/his1::tetR-FRT arg4/arg4 IRO1/iro1Δ::λimm^{434} URA3/ura3Δ::λimm^{434}*	JKC1450 transformed with KpnI/BsiWI digested pJK1372 to delete the 1st allele of *PHO87*.	This work
JKC2712	JKC2599	*pho84::HIS1/pho84::ARG4 pho87::uPAM-FRT/pho87::uPAM-FRT PHO89/pho89::uPAM-FRT-FLP-NAT1 his1/his1::tetR-FRT arg4/arg4 IRO1/iro1Δ::λimm^{434}*	JKC2599 transformed with KpnI/BsiWI digested pJK1384 to delete the 1st allele of *PHO89*.	This work

		URA3/ura3Δ::λimm^{434}		
JKC2790	JKC2773	*pho84::HIS1/pho84::ARG4*<br>*pho89::uPAM-FRT/pho89::uPAM-FRT*<br>*fgr2::uPAM-FRT/fgr2::uPAM-FRT*<br>*PHO87/pho87::FRT-FLP-NAT1-tetO-PHO87*<br>*his1/his1::tetR-FRT arg4/arg4 IRO1/iro1Δ::*λimm^{434}<br>*URA3/ura3Δ::*λimm^{434}	JKC2773 transformed with KpnI/NcoI digested pJK1375 to have one of the alleles of *PHO87* under the *tetO* control	This work
JKC2652	JKC2638	*pho87::uPAM-FRT/pho87::uPAM-FRT*<br>*PHO89/pho89::uPAM-FRT*<br>*HIS1/his1::tetR-FRT*	JKC2638 *NAT1* flipped out	This work
JKC2566	JKC2545	*pho84::HIS1/pho84::ARG4*<br>*PHO89/pho89::uPAM-FRT*<br>*his1/his1::tetR-FRT arg4/arg4 IRO1/iro1Δ::*λimm^{434}<br>*URA3/ura3Δ::*λimm^{434}	JKC2545 *NAT1* flipped out	This work
JKC2554	JKC2539	*pho84::HIS1/pho84::ARG4*<br>*PHO87/pho87::uPAM-FRT*<br>*his1/his1::tetR-FRT arg4/arg4 IRO1/iro1Δ::*λimm^{434}<br>*URA3/ura3Δ::*λimm^{434}	JKC2539 *NAT1* flipped out	This work
JKC2718	JKC2712	*pho84::HIS1/pho84::ARG4*<br>*pho87::uPAM-FRT/pho87::uPAM-FRT*<br>*PHO89/pho89::uPAM-FRT*<br>*his1/his1::tetR-FRT arg4/arg4 IRO1/iro1Δ::*λimm^{434}<br>*URA3/ura3Δ::*λimm^{434}	JKC2712 *NAT1* flipped out	This work
JKC2793	JKC2790	*pho84::HIS1/pho84::ARG4*<br>*pho89::uPAM-FRT/pho89::uPAM-FRT*<br>*fgr2::uPAM-FRT/fgr2::uPAM-FRT*<br>*PHO87/pho87::FRT-tetO-PHO87*<br>*his1/his1::tetR-FRT arg4/arg4 IRO1/iro1Δ::*λimm^{434}<br>*URA3/ura3Δ::*λimm^{434}	JKC2790 *NAT1* flipped out	This work
JKC2664	JKC2652	*pho87::uPAM-FRT/pho87::uPAM-FRT*<br>*pho89::uPAM-FRT/pho89::uPAM-FRT-FLP-NAT1*<br>*HIS1/his1::tetR-FRT*	JKC2652 transformed with KpnI/BsiWI digested pJK1481 to delete the 2nd allele of *PHO89*.	This work
JKC2755	JKC2718	*pho84::HIS1/pho84::ARG4*<br>*pho87::uPAM-FRT/pho87::uPAM-FRT*<br>*pho89::uPAM-FRT/pho89::uPAM-FRT-FLP-NAT1*<br>*his1/his1::tetR-FRT arg4/arg4 IRO1/iro1Δ::*λimm^{434}<br>*URA3/ura3Δ::*λimm^{434}	JKC2718 transformed with KpnI/BsiWI digested pJK1481 to delete the 2nd allele of *PHO89*.	This work
JKC2799	JKC2793	*pho84::HIS1/pho84::ARG4*<br>*pho89::uPAM-FRT/pho89::uPAM-FRT*<br>*fgr2::uPAM-FRT/fgr2::uPAM-FRT*<br>*pho87::FRT-tetO-PHO87/pho87::uPAM-FRT-FLP-NAT1*<br>*his1/his1::tetR-FRT arg4/arg4 IRO1/iro1Δ::*λimm^{434}<br>*URA3/ura3Δ::*λimm^{434}	JKC2793 transformed with KpnI/BsiWI digested pJK1372 to delete the wildtype allele of *PHO87*.	This work
JKC2800	JKC2793	Same as JKC2799, different isolate	Same as JKC2799, different isolate	This work
JKC2679	JKC2664	*pho87::uPAM-FRT/pho87::uPAM-FRT*<br>*pho89::uPAM-FRT/pho89::uPAM-FRT*<br>*HIS1/his1::tetR-FRT*	JKC2664 *NAT1* flipped out	This work
JKC2758	JKC2755	*pho84::HIS1/pho84::ARG4*<br>*pho87::uPAM-FRT/pho87::uPAM-FRT*<br>*pho89::uPAM-FRT/pho89::uPAM-FRT*<br>*his1/his1::tetR-FRT arg4/arg4 IRO1/iro1Δ::*λimm^{434}<br>*URA3/ura3Δ::*λimm^{434}	JKC2755 *NAT1* flipped out	This work
JKC2804	JKC2799	*pho84::HIS1/pho84::ARG4*<br>*pho89::uPAM-FRT/pho89::uPAM-FRT*<br>*fgr2::uPAM-FRT/fgr2::uPAM-FRT*<br>*pho87::FRT-tetO-PHO87/pho87::uPAM-FRT*<br>*his1/his1::tetR-FRT arg4/arg4 IRO1/iro1Δ::*λimm^{434}<br>*URA3/ura3Δ::*λimm^{434}	JKC2799 *NAT1* flipped out	This work
JKC2806	JKC2800	Same as JKC2804, flip-out from a different isolate	JKC2800 *NAT1* flipped out	This work
JKC2542	JKC915	*PHO89/pho89::uPAM-FRT-FLP-NAT1*<br>*HIS1/his1::tetR-FRT*	JKC915 transformed with KpnI/BsiWI digested pJK1384 to delete the 1st allele of *PHO89*.	This work
JKC2632	JKC915	*FGR2/fgr2::uPAM-FRT-FLP-NAT1*<br>*HIS1/his1::tetR-FRT*	JKC915 transformed with KpnI/BsiWI digested pJK1485 to delete the 1st allele of *FGR2*.	This work
JKC2734	JKC2679	*pho87::uPAM-FRT/pho87::uPAM-FRT*<br>*pho89::uPAM-FRT/pho89::uPAM-FRT*<br>*FGR2/fgr2::uPAM-FRT-FLP-NAT1*<br>*HIS1/his1::tetR-FRT*	JKC2679 transformed with KpnI/BsiWI digested pJK1485 to delete the 1st allele of *FGR2*.	This work
JKC2809	JKC2758	*pho84::HIS1/pho84::ARG4*<br>*pho87::uPAM-FRT/pho87::uPAM-FRT*<br>*pho89::uPAM-FRT/pho89::uPAM-FRT*<br>*FGR2/fgr2::uPAM-FRT-FLP-NAT1*	JKC2758 transformed with KpnI/BsiWI digested pJK1485 to delete the 1st allele of *FGR2*.	This work

		his1/his1::tetR-FRT arg4/arg4 IRO1/iro1Δ::λimm^{434}<br>*URA3/ura3Δ::λimm*^{434}		
JKC2915	JKC2804	*pho84::HIS1/pho84::ARG4*<br>*pho89::uPAM-FRT/pho89::uPAM-FRT*<br>*fgr2::uPAM-FRT/fgr2::uPAM-FRT*<br>*pho87::FRT-tetO-PHO87/pho87::uPAM-FRT*<br>*GIT2-4/git2-4::uPAM-FRT-FLP-NAT1*<br>*his1/his1::tetR-FRT arg4/arg4 IRO1/iro1Δ::λimm*^{434}<br>*URA3/ura3Δ::λimm*^{434}	JKC2804 transformed with KpnI/BsiWI digested pJK1543 to delete the 1st allele of *GIT2-4*.	This work
JKC2917	JKC2806	Same as JKC2915, derived from JKC2806.	JKC2806 transformed with KpnI/BsiWI digested pJK1543 to delete the 1st allele of *GIT2-4*.	This work
JKC2560	JKC2542	*PHO89/pho89::uPAM-FRT*<br>*HIS1/his1::tetR-FRT*	JKC2542 *NAT1* flipped out	This work
JKC2641	JKC2632	*FGR2/fgr2::uPAM-FRT*<br>*HIS1/his1::tetR-FRT*	JKC2632 *NAT1* flipped out	This work
JKC2749	JKC2734	*pho87::uPAM-FRT/pho87::uPAM-FRT*<br>*pho89::uPAM-FRT/pho89::uPAM-FRT*<br>*FGR2/fgr2::uPAM-FRT*<br>*HIS1/his1::tetR-FRT*	JKC2734 *NAT1* flipped out	This work
JKC2812	JKC2809	*pho84::HIS1/pho84::ARG4*<br>*pho87::uPAM-FRT/pho87::uPAM-FRT*<br>*pho89::uPAM-FRT/pho89::uPAM-FRT*<br>*FGR2/fgr2::uPAM-FRT*<br>*his1/his1::tetR-FRT arg4/arg4 IRO1/iro1Δ::λimm*^{434}<br>*URA3/ura3Δ::λimm*^{434}	JKC2809 *NAT1* flipped out	This work
JKC2826	JKC2812	*pho84::HIS1/pho84::ARG4*<br>*pho87::uPAM-FRT/pho87::uPAM-FRT*<br>*pho89::uPAM-FRT/pho89::uPAM-FRT*<br>*fgr2::uPAM-FRT/fgr2::uPAM-FRT-FLP-NAT1*<br>*his1/his1::tetR-FRT arg4/arg4 IRO1/iro1Δ::λimm*^{434}<br>*URA3/ura3Δ::λimm*^{434}	JKC2812 transformed with KpnI/BsiWI digested pJK1488 to delete the 2nd allele of *FGR2*.	This work
JKC2926	JKC2915	*pho84::HIS1/pho84::ARG4*<br>*pho89::uPAM-FRT/pho89::uPAM-FRT*<br>*fgr2::uPAM-FRT/fgr2::uPAM-FRT*<br>*pho87::FRT-tetO-PHO87/pho87::uPAM-FRT*<br>*GIT2-4/git2-4::uPAM-FRT*<br>*his1/his1::tetR-FRT arg4/arg4 IRO1/iro1Δ::λimm*^{434}<br>*URA3/ura3Δ::λimm*^{434}	JKC2915 *NAT1* flipped out	This work
JKC2930	JKC2917	Same as JKC2926, derived from JKC2806.	JKC2917 *NAT1* flipped out	This work
JKC2575	JKC2560	*pho89::uPAM-FRT/pho89::uPAM-FRT-FLP-NAT1*<br>*HIS1/his1::tetR-FRT*	JKC2560 transformed with KpnI/BsiWI digested pJK1481 to delete the 2nd allele of *PHO89*.	This work
JKC2658	JKC2641	*fgr2::uPAM-FRT/fgr2::uPAM-FRT-FLP-NAT1*<br>*HIS1/his1::tetR-FRT*	JKC2641 transformed with KpnI/BsiWI digested pJK1488 to delete the 2nd allele of *FGR2*.	This work
JKC2772	JKC2749	*pho87::uPAM-FRT/pho87::uPAM-FRT*<br>*pho89::uPAM-FRT/pho89::uPAM-FRT*<br>*fgr2::uPAM-FRT/fgr2::uPAM-FRT-FLP-NAT1*<br>*HIS1/his1::tetR-FRT*	JKC2749 transformed with KpnI/BsiWI digested pJK1488 to delete the 2nd allele of *FGR2*.	This work
JKC2766	JKC2737	*pho84::HIS1/pho84::ARG4*<br>*pho89::uPAM-FRT/pho89::uPAM-FRT*<br>*fgr2::uPAM-FRT/fgr2::uPAM-FRT-FLP-NAT1*<br>*his1/his1::tetR-FRT arg4/arg4 IRO1/iro1Δ::λimm*^{434}<br>*URA3/ura3Δ::λimm*^{434}	JKC2737 transformed with KpnI/BsiWI digested pJK1488 to delete the 2nd allele of *FGR2*.	This work
JKC2769	JKC2743	*pho84::HIS1/pho84::ARG4*<br>*pho87::uPAM-FRT/pho87::uPAM-FRT*<br>*fgr2::uPAM-FRT/fgr2::uPAM-FRT-FLP-NAT1*<br>*his1/his1::tetR-FRT arg4/arg4 IRO1/iro1Δ::λimm*^{434}<br>*URA3/ura3Δ::λimm*^{434}	JKC2743 transformed with KpnI/BsiWI digested pJK1488 to delete the 2nd allele of *FGR2*.	This work
JKC2743	JKC2731	*pho84::HIS1/pho84::ARG4*<br>*pho87::uPAM-FRT/pho87::uPAM-FRT*<br>*FGR2/fgr2::uPAM-FRT*<br>*his1/his1::tetR-FRT arg4/arg4 IRO1/iro1Δ::λimm*^{434}<br>*URA3/ura3Δ::λimm*^{434}	JKC2731 *NAT1* flipped out	This work
JKC2731	JKC2599	*pho84::HIS1/pho84::ARG4*<br>*pho87::uPAM-FRT/pho87::uPAM-FRT*<br>*FGR2/fgr2::uPAM-FRT-FLP-NAT1*<br>*his1/his1::tetR-FRT arg4/arg4 IRO1/iro1Δ::λimm*^{434}<br>*URA3/ura3Δ::λimm*^{434}	JKC2599 transformed with KpnI/BsiWI digested pJK1485 to delete the 1st allele of *FGR2*.	This work
JKC2844	JKC2812	Same as JKC2826.	Same as JKC2826, different isolate.	This work
JKC2957	JKC2926	*pho84::HIS1/pho84::ARG4*<br>*pho89::uPAM-FRT/pho89::uPAM-FRT*	JKC2926 transformed with KpnI/BsiWI digested pJK1545 to	This work

		fgr2::uPAM-FRT/fgr2::uPAM-FRT pho87::FRT-tetO-PHO87/pho87::uPAM-FRT git2-4::uPAM-FRT/git2-4::uPAM-FRT-FLP-NAT1 his1/his1::tetR-FRT arg4/arg4 IRO1/iro1Δ::λimm^{434} *URA3/ura3Δ::λimm*^{434}	delete the 2nd allele of *GIT2-4*.	
JKC2958	JKC2926	Same as JKC2957.	Same as JKC2957, different isolate.	This work
JKC2961	JKC2930	Same as JKC2957, derived from JKC2930	Same as JKC2957, derived from JKC2930	This work
JKC2585 "*pho89-/-*"	JKC2575	*pho89::uPAM-FRT/pho89::uPAM-FRT HIS1/his1::tetR-FRT*	JKC2575 *NAT1* flipped out	This work
JKC2667 "*fgr2-/-*"	JKC2658	*fgr2::uPAM-FRT/fgr2::uPAM-FRT HIS1/his1::tetR-FRT*	JKC2658 *NAT1* flipped out	This work
JKC2788 "Pho84-A"	JKC2772	*pho87::uPAM-FRT/pho87::uPAM-FRT pho89::uPAM-FRT/pho89::uPAM-FRT fgr2::uPAM-FRT/fgr2::uPAM-FRT HIS1/his1::tetR-FRT*	JKC2772 *NAT1* flipped out	This work
JKC2777 "Pho87-A"	JKC2766	*pho84::HIS1/pho84::ARG4 pho89::uPAM-FRT/pho89::uPAM-FRT fgr2::uPAM-FRT/fgr2::uPAM-FRT his1/his1::tetR-FRT arg4/arg4 IRO1/iro1Δ::λimm*^{434} *URA3/ura3Δ::λimm*^{434}	JKC2766 *NAT1* flipped out	This work
JKC2783 "Pho89-A"	JKC2769	*pho84::HIS1/pho84::ARG4 pho87::uPAM-FRT/pho87::uPAM-FRT fgr2::uPAM-FRT/fgr2::uPAM-FRT his1/his1::tetR-FRT arg4/arg4 IRO1/iro1Δ::λimm*^{434} *URA3/ura3Δ::λimm*^{434}	JKC2769 *NAT1* flipped out	This work
JKC2830 "Q-L1"	JKC2826	*pho84::HIS1/pho84::ARG4 pho87::uPAM-FRT/pho87::uPAM-FRT pho89::uPAM-FRT/pho89::uPAM-FRT fgr2::uPAM-FRT/fgr2::uPAM-FRT his1/his1::tetR-FRT arg4/arg4 IRO1/iro1Δ::λimm*^{434} *URA3/ura3Δ::λimm*^{434}	JKC2826 *NAT1* flipped out	This work
JKC2831 "Q-"	JKC2826	Same as JKC2830, different flip-out isolate	JKC2826 *NAT1* flipped out	This work
JKC2858 "Q-"	JKC2844	Same as JKC2830, flip-out from JKC2844	JKC2844 *NAT1* flipped out	This work
JKC2859 "Q-"	JKC2844	Same as JKC2830, flip-out from JKC2844 Different isolate from JKC2858	JKC2844 *NAT1* flipped out	This work
JKC2845	JKC2812	Same as JKC2826.	Same as JKC2826, different isolate.	This work
JKC2860 "Q-L2"	JKC2845	Same as JKC2830, flip-out from JKC2845	JKC2845 *NAT1* flipped out	This work
JKC2861 "Q-"	JKC2845	Same as JKC2830, flip-out from JKC2845	JKC2845 *NAT1* flipped out	This work
JKC2967 "Septuple mutant *tetO-PHO87*"	JKC2957	*pho84::HIS1/pho84::ARG4 pho89::uPAM-FRT/pho89::uPAM-FRT fgr2::uPAM-FRT/fgr2::uPAM-FRT pho87::FRT-tetO-PHO87/pho87::uPAM-FRT git2-4::uPAM-FRT/git2-4::uPAM-FRT his1/his1::tetR-FRT arg4/arg4 IRO1/iro1Δ::λimm*^{434} *URA3/ura3Δ::λimm*^{434}	JKC2957 *NAT1* flipped out	This work
JKC2969 "Septuple mutant *tetO-PHO87*"	JKC2958	Same as JKC2967, flip-out from JKC2958.	JKC2958 *NAT1* flipped out	This work
JKC2973 "Septuple mutant *tetO-PHO87*"	JKC2961	Same as JKC2967, derived from JKC2930	JKC2961 *NAT1* flipped out	This work

**uPAM* is an artificially designed sequence (universal-PAM sequence) in our *FLP-NAT1* cassette that we use in other work for CRISPR guide RNA recognition; not used in this work.

Construction of strains with multiple mutations

Construction of the Pho84-A strain (*pho87-/- pho89-/- fgr2-/-* triple mutant):

The Pho84-Alone (Pho84-A) strain JKC2788 was constructed as follows: wild type strain JKC915 [3] was transformed with pJK1372 and pJK1479 to sequentially delete two alleles of *PHO87*, resulting in JKC2581 (*pho87-/-*). JKC2581 (*pho87-/-*) was then transformed with pJK1384 and pJK1481 to sequentially delete two alleles of *PHO89*, resulting in JKC2679 (*pho87-/- pho89-/-*).

JKC2679 (*pho87-/- pho89-/-*) was eventually transformed with pJK1485 and pJK1488 to sequentially delete two alleles of *FGR2*, resulting in JKC2788 (*pho87-/- pho89-/- fgr2-/-*).

Construction of the Pho87-A strain (*pho84-/- pho89-/- fgr2-/-* triple mutant):

The Pho87-Alone (Pho87-A) strain JKC2777 was constructed as follows: JKC1450 (*pho84-/-*) [4] was transformed with pJK1384 and pJK1481 to sequentially delete two alleles of *PHO89*, resulting in JKC2592 (*pho84-/- pho89-/-*). JKC2592 (*pho84-/- pho89-/-*) was eventually transformed with pJK1485 and pJK1488 to sequentially delete two alleles of *FGR2*, resulting in JKC2777 (*pho84-/- pho89-/- fgr2-/-*).

Construction of the Pho89-A strain (*pho84-/- pho87-/- fgr2-/-* triple mutant):

The Pho89-Alone (Pho89-A) strain JKC2783 was constructed as follows: JKC1450 (*pho84-/-*) [4] was transformed with pJK1372 and pJK1479 to sequentially delete two alleles of *PHO87*, resulting in JKC2599 (*pho84-/- pho87-/-*). JKC2599 (*pho84-/- pho87-/-*) was eventually transformed with pJK1485 and pJK1488 to sequentially delete two alleles of *FGR2*, resulting in JKC2783 (*pho84-/- pho87-/- fgr2-/-*).

Construction of the Fgr2-A strain (*pho84-/- pho87-/- pho89-/-* triple mutant):

The Fgr2-Alone (Fgr2-A) strain JKC2758 was constructed as follows: JKC1450 (*pho84-/-*) [4] was transformed with pJK1372 and pJK1479 to sequentially delete two alleles of *PHO87*, resulting in JKC2599 (*pho84-/- pho87-/-*). JKC2599 (*pho84-/- pho87-/-*) was eventually transformed with pJK1384 and pJK1481 to sequentially delete two alleles of *PHO89*, resulting in JKC2758 (*pho84-/- pho87-/- pho89-/-*).

Construction of quadruple mutants:

Using the quadruple mutant strain JKC2830 as an example, JKC1450 (*pho84-/-*) [4] was transformed with pJK1372 and pJK1479 to sequentially delete two alleles of *PHO87*, resulting in JKC2599 (*pho84-/- pho87-/-*). JKC2599 (*pho84-/- pho87-/-*) was then transformed with pJK1384 and pJK1481 to sequentially delete two alleles of *PHO89*, resulting in JKC2758 (*pho84-/- pho87-/- pho89-/-*). JKC2758 (*pho84-/- pho87-/- pho89-/-*) was eventually transformed with pJK1485 and pJK1488 to sequentially delete two alleles of *FGR2*, resulting in JKC2830 (*pho84-/- pho87-/- pho89-/- fgr2-/-*).

Construction of septuple mutants:

Using the septuple mutant strain JKC2969 as an example, JKC1450 (*pho84-/-*) [4] was transformed with pJK1384 and pJK1481 to sequentially delete two alleles of *PHO89*, resulting in JKC2592 (*pho84-/- pho89-/-*). JKC2592 (*pho84-/- pho89-/-*) was then transformed with pJK1485 and pJK1488 to sequentially delete two alleles of *FGR2*, resulting in JKC2773 (*pho84-/- pho89-/- fgr2-/-*). JKC2773 (*pho84-/- pho89-/- fgr2-/-*) was first transformed with pJK1375 to have one allele of *PHO87* under *tetO* control, then transformed with pJK1372 to delete the WT allele of *PHO87*, resulting in JKC2804 (*pho84-/- pho89-/- fgr2-/- pho87/tetO-PHO87*). JKC2804 (*pho84-/- pho89-/- fgr2-/- pho87/tetO-PHO87*) was eventually transformed with pJK1543 and pJK1545 to sequentially delete two alleles of *GIT2-4*, resulting in JKC2969 (*pho84-/- pho89-/- fgr2-/- PHO87-/tetO git2-4-/-*).

References

1. Fonzi WA, Irwin MY. Isogenic strain construction and gene mapping in Candida albicans. Genetics. 1993;134(3):717-28. Epub 1993/07/01. PubMed PMID: 8349105; PubMed Central PMCID: PMC1205510.
2. Noble SM, Johnson AD. Strains and strategies for large-scale gene deletion studies of the diploid human fungal pathogen Candida albicans. Eukaryotic cell. 2005;4(2):298-309. Epub 2005/02/11. doi: 10.1128/EC.4.2.298-309.2005. PubMed PMID: 15701792; PubMed Central PMCID: PMC549318.
3. Shen J, Cowen LE, Griffin AM, Chan L, Köhler JR. The Candida albicans pescadillo homolog is required for normal hypha-to-yeast morphogenesis and yeast proliferation. Proceedings of the National Academy of Sciences of the United States of America. 2008;105(52):20918-23. Epub 2008/12/17. doi: 10.1073/pnas.0809147105. PubMed PMID: 19075239; PubMed Central PMCID: PMC2634893.
4. Liu NN, Flanagan PR, Zeng J, Jani NM, Cardenas ME, Moran GP, et al. Phosphate is the third nutrient monitored by TOR in Candida albicans and provides a target for fungal-specific indirect TOR inhibition. Proceedings of the National Academy of Sciences of the United States of America. 2017. Epub 2017/06/02. doi: 10.1073/pnas.1617799114. PubMed PMID: 28566496.
