# Supplementary material for: Candida albicans’ inorganic phosphate transport and evolutionary adaptation to phosphate scarcity
Source: PLoS Genet. 2024 Aug 13;20(8):e1011156. doi: 10.1371/journal.pgen.1011156 (PMC11343460; doi:10.1371/journal.pgen.1011156)
Supplement: S3 Table — (PDF) [file pgen.1011156.s004.pdf]

S3 Table. Plasmids used in this study.

Plasmid	Description	Source (Reference)
pJK1000	*FLP-NAT1 tetO-PES1* construct, vector backbone is pLitmus28 (New England Biolabs)	[1]
pJK1372	*FLP-NAT1 pho87* deletion construct, derived from pJK1364. Product of fjk1846 and r1862 using SC5314 genomic DNA as template was ligated into pJK1364 using KpnI/ApaI sites.	[2]
pJK1375	*FLP-NAT1 tetO-PHO87* construct, derived from pJK1000. Product of fjk1854 and rjk1855 using SC5314 genomic DNA as template and product of fjk1856 and rjk1857 using SC5314 genomic DNA as template were ligated into pJK1000 using KpnI/ApaI and SacII/NcoI sites, respectively.	This work
pJK1384	*FLP-NAT1 pho89* deletion construct, derived from pJK1372. Product of fjk1869 and rjk1870 using SC5314 genomic DNA as template and product of fjk1871 and rjk1872 using SC5314 genomic DNA as template were ligated into pJK1372 using KpnI/AscI and NotI/BsiWI sites, respectively.	This work
pJK1479	*FLP-NAT1 pho87* 2nd allele deletion construct, derived from pJK1372. Product of fjk2032 and rjk2033 using SC5314 genomic DNA as template was ligated into pJK1372 using KpnI/AscI sites.	This work
pJK1481	*FLP-NAT1 pho89* 2nd allele deletion construct, derived from pJK1372. Product of fjk2034 and rjk2035 using SC5314 genomic DNA as template and product of fjk1871 and rjk1872 using SC5314 genomic DNA as template were ligated into pJK1372 using KpnI/AscI and NotI/BsiWI sites, respectively.	This work
pJK1485	*FLP-NAT1 fgr2* deletion construct, derived from pJK1372. Product of fjk2037 and rjk2038 using SC5314 genomic DNA as template and product of fjk2041 and rjk2042 using SC5314 genomic DNA as template were ligated into pJK1372 using KpnI/AscI and NotI/BsiWI sites, respectively.	This work
pJK1488	*FLP-NAT1 fgr2* 2nd allele deletion construct, derived from pJK1372. Product of fjk2039 and rjk2040 using SC5314 genomic DNA as template and product of fjk2041 and rjk2042 using SC5314 genomic DNA as template were ligated into pJK1372 using KpnI/AscI and NotI/BsiWI sites, respectively.	This work
pJK1543	*FLP-NAT1 git2-4* deletion construct, derived from pJK1372. Product of fjk2197 and rjk2198 using SC5314 genomic DNA as template and product of fjk2201 and rjk2202 using SC5314 genomic DNA as template were ligated into pJK1372 using KpnI/AscI and NotI/BsiWI sites, respectively.	This work
pJK1545	*FLP-NAT1 git2-4* 2nd allele deletion construct, derived from pJK1372. Product of fjk2199 and rjk2200 using SC5314 genomic DNA as template and product of fjk2201 and rjk2202 using SC5314 genomic DNA as template were ligated into pJK1372 using KpnI/AscI and NotI/BsiWI sites, respectively.	This work

References

1. Shen J, Cowen LE, Griffin AM, Chan L, Köhler JR. The Candida albicans pescadillo homolog is required for normal hypha-to-yeast morphogenesis and yeast proliferation. Proceedings of the National Academy of Sciences of the United States of America. 2008;105(52):20918-23. Epub 2008/12/17. doi: 10.1073/pnas.0809147105. PubMed PMID: 19075239; PubMed Central PMCID: PMC2634893.
2. Liu NN, Acosta-Zaldivar M, Qi W, Diray-Arce J, Walker LA, Kottom TJ, et al. Phosphoric Metabolites Link Phosphate Import and Polysaccharide Biosynthesis for Candida albicans Cell Wall Maintenance. mBio. 2020;11(2). Epub 2020/03/19. doi: 10.1128/mBio.03225-19. PubMed PMID: 32184254; PubMed Central PMCID: PMCPMC7078483.
