# Supplementary material for: Candida albicans’ inorganic phosphate transport and evolutionary adaptation to phosphate scarcity
Source: PLoS Genet. 2024 Aug 13;20(8):e1011156. doi: 10.1371/journal.pgen.1011156 (PMC11343460; doi:10.1371/journal.pgen.1011156)
Supplement: S4 Table — (PDF) [file pgen.1011156.s005.pdf]

S4 Table. Oligonucleotides used in this study.

Primer name	Purpose	Sequence 5' to 3' (lower cases - restriction enzyme recognition sites)
fjk1854	Forward oligo to amplify the *tetO-PHO87* construct upstream homologous sequence Also used to verify the 5'end of *pho87* deletion mutant	CATCCGggtaccCAATAGAGCGGGAATGGAAA
rjk1855	Reverse oligo to amplify the *tetO-PHO87* construct upstream homologous sequence	GATCgggcccTCAATTTCACCCATCAAAAACA
fjk1856	Forward oligo to amplify the *tetO-PHO87* construct downstream homologous sequence	GGATCCccgcggATGAAGTTTTCTCATTCATTG
rjk1857	Reverse oligo to amplify the *tetO-PHO87* construct downstream homologous sequence	CTCATGccatggGATTTCTAAATCACTTTCACTACCATAA
fjk1877	Forward oligo to verify the 5'end of *tetO-PHO87* integration	GCTTGCTGGTTTAACCTGAG
rjk1878	Reverse oligo to verify the 3'end of *tetO-PHO87* integration	GTGTCATGTTCACCATTGGA
fjk1869	Forward oligo to amplify the *pho89* deletion construct upstream homologous sequence	CATCCGggtaccTTGCAATTATTTTTCTTGTCCAAA
rjk1870	Reverse oligo to amplify the *pho89* deletion construct upstream homologous sequence	GATggcgcgccTGTATATATTTGAATTTATTTGTTGTTG
fjk1871	Forward oligo to amplify the *pho89* deletion construct downstream homologous sequence	AAGGTAAGCAgcggccgcACGTTGTTTGTGGTTTCAATTTAG
rjk1872	Reverse oligo to amplify the *pho89* deletion construct downstream homologous sequence	CTCATGcgtacgCCTTGGCATAGCATTAGTAATCA
fjk1873	Forward oligo to verify the 5'end of *pho89* deletion mutant	CATCCGggtaccCGACAAACATGCTTCCTTGA
rjk1885	Reverse oligo to verify the 3'end of *pho89* deletion mutant	CGACACTTCTTGGATTTGCT
fjk2032	Forward oligo to amplify the *pho87* deletion-2nd allele construct upstream homologous sequence	CATCCGggtaccATGAAGTTTTCTCATTCATTGAAATTTAATGC
rjk2033	Reverse oligo to amplify the *pho87* deletion-2nd allele construct upstream homologous sequence	GATggcgcgccCATTGGTGTTGTTATTTCTTTTGAA
rjk1339	Reverse oligo to verify the 5'end of *integration of 'FLP-NAT1'* cassette containing constructs	TGGTGTGTTGTTGACAGGCAAC
fjk490	Forward oligo to verify the 3'end integration of '*FLP-NAT1'* cassette containing constructs	TCAAGGAGGGTATTCTGGGC
fjk1835	Forward oligo to verify the 3'end integration of '*FLP-NAT1-tetO'* constructs	TGTCGTTTCTGATGGGCTTT
rjk1879	Reverse oligo to verify the 3'end of *pho87* deletion mutant	AACAACAACCACAACCACAA
fjk2034	Forward oligo to amplify the *pho89* deletion-2nd allele construct upstream homologous sequence	CATCCGggtaccATGGCTTTACATCAATTTGATTATTTGTTTG
rjk2035	Reverse oligo to amplify the *pho89* deletion-2nd allele construct upstream homologous sequence	GATggcgcgccGTCATAGTCAACATCAACACAGCAG
fjk2037	Forward oligo to amplify the *fgr2* deletion construct upstream homologous sequence	CATCAAggtaccAACTCTATTTCTCGAAGCTGTCAAA
rjk2038	Reverse oligo to amplify the *fgr2* deletion construct upstream homologous sequence	GATggcgcgccAGACTCAATTACGAAACACAAGACC
fjk2039	Forward oligo to amplify the *fgr2* deletion-2nd allele construct upstream homologous sequence	CATCAAggtaccTCTCACACGTTCCAAATAAGAAAAC
rjk2040	Reverse oligo to amplify the *fgr2* deletion-2nd allele construct upstream homologous sequence	GATggcgcgccTTTCAGTTACAGACGGAATGAATAA
fjk2041	Forward oligo to amplify the *fgr2* deletion construct downstream homologous sequence	TTGGTAAGCAgcggccgcATTTAATATCCAACTTAGCTCAAATAA
rjk2042	Reverse oligo to amplify the *fgr2* deletion construct downstream homologous sequence	CTCATGcgtacgCCCTTTGAAGATATATTTGATGAAACC
fjk2043	Forward oligo to verify the 5'end of *fgr2* deletion mutant	TATAACCTAGCAGAATAGCCGATTG
rjk2044	Reverse oligo to verify the 3'end of *fgr2* deletion mutant	ACGTATTGGTTGAATTTTGGAGTAG
fjk2197	Forward oligo to amplify the *git2-4* deletion construct upstream homologous sequence	CATCAAggtaccATTTAGGCTGCAAAAAGAGAAAAAT
rjk2198	Reverse oligo to amplify the *git2-4* deletion construct upstream homologous sequence	GATggcgcgccTGCTCTGATTAATCTTCGACCTAGT
fjk2199	Forward oligo to amplify the *git2-4* deletion-2nd allele construct upstream homologous sequence	CATCAAggtaccTCAATTAATAGTCTTTGCCATAAACA
rjk2200	Reverse oligo to amplify the *git2-4* deletion-2nd allele construct upstream homologous sequence	GATggcgcgccCCATGTTATTATTTGTTTGACTTGTTAGG
fjk2201	Forward oligo to amplify the *git2-4* deletion construct downstream homologous sequence	TTGGTAAGCAgcggccgcGGAGGTTCAACTTTGCAGGT

rjk2202	Reverse oligo to amplify the *git2-4* deletion construct downstream homologous sequence	CTCATGcgtacgTTGCCGAAGTGGGTTTGTAT
fjk2203	Forward oligo to verify the 5'end of *git2-4* deletion mutant	ATACACACACTCCCAAAAACTCATT
rjk2204	Reverse oligo to verify the 3'end of *git2-4* deletion mutant	GCCACCAAGTAGGTTTGGAA
