# Supplementary material for: Candida albicans’ inorganic phosphate transport and evolutionary adaptation to phosphate scarcity
Source: PLoS Genet. 2024 Aug 13;20(8):e1011156. doi: 10.1371/journal.pgen.1011156 (PMC11343460; doi:10.1371/journal.pgen.1011156)
Supplement: S5 Table — (PDF) [file pgen.1011156.s006.pdf]

S5 Table. Antibodies used in this study.

Purpose	Antigen recognized	Species	Source or Reference
loading control	tubulin	rat	Abcam, cat. # ab6161
P-S6	phospho (S/T)-Akt substrate	rabbit	Cell Signaling Technology, cat. # 9611
secondary	rat IgG	goat	Santa Cruz Biotechnology, cat. #97057
secondary	rabbit IgG	goat	Cell Signaling Technology, cat. #7074S
